# Seasonal bacterial community succession in four typical wastewater treatment plants: correlations between core microbes and process performance

**DOI:** 10.1038/s41598-018-22683-1

**Published:** 2018-03-15

**Authors:** Bo Zhang, Quanwei Yu, Guoqi Yan, Hubo Zhu, Xiang yang Xu, Liang Zhu

**Affiliations:** 1 Department of Environmental Engineering, Zhejiang University, Hangzhou, 310058 China; 2 Zhejiang Province Key Laboratory for Water Pollution Control and Environmental Safety, Hangzhou, 310058 China; 3 Hangzhou Drainage Co., LTD, East City Water Treatment Branch, Hangzhou, 310018 China; 4 Shangyu Wastewater Treatment Plant, Shangyu, 312369 China

## Abstract

To understand the seasonal variation of the activated sludge (AS) bacterial community and identify core microbes in different wastewater processing systems, seasonal AS samples were taken from every biological treatment unit within 4 full-scale wastewater treatment plants. These plants adopted A2/O, A/O and oxidation ditch processes and were active in the treatment of different types and sources of wastewater, some domestic and others industrial. The bacterial community composition was analyzed using high-throughput sequencing technology. The correlations among microbial community structure, dominant microbes and process performance were investigated. Seasonal variation had a stronger impact on the AS bacterial community than any variation within different wastewater treatment system. Facing seasonal variation, the bacterial community within the oxidation ditch process remained more stable those in either the A2/O or A/O processes. The core genera in domestic wastewater treatment systems were *Nitrospira*, *Caldilineaceae*, *Pseudomonas* and *Lactococcus*. The core genera in the textile dyeing and fine chemical industrial wastewater treatment systems were *Nitrospira*, *Thauera* and *Thiobacillus*.

## Introduction

Municipal wastewater treatment plants (WWTPs) are vitally important units working to protect the ecology of river environments. The various treatment processes involved in these WWTPs are determined by numerous factors including influent characteristic, effluent requirement, WWTP size, local temperature, land-use fees, geology, electrovalence, and others. Different processes have their own specific advantages and disadvantages with various differing specialties and application conditions. For example, a simple structured oxidation ditch is mainly used to remove carbon under aerobic conditions^1^. More advanced configurations, such as anaerobic/oxic process (A/O), anaerobic/anoxic/oxic processe (A2/O), are used for nitrogen and/or phosphate removal^2^. In some cases, anaerobic hydrolysis processes are grouped as pretreatment options to be used for reducing the quantity of organic and improving the biodegradability of influent^3,4^. Despite these differences, in all cases activated sludge (AS) is the main contributor to the removal of the pollutants^5^ and a positive association is therefore expected between the function and AS taxonomic richness.

However, for full-scale WWTPs, the relationship between the performance and operational parameters of the plants and the resulting bacterial community diversity received limited attention^6^. Zhang *et al*. studied the effects of geographic variation on AS population structure in various WWTPs. Their paper demonstrated that some core genera were common between samples separated by large geographical distances^7^. Yang *et al*. analyzed the AS bacterial community of two WWTPs in northern China which adopted A2/0 processes. His team found that *Proteobacteria* was the most abundant phylogenetic group, followed by *Bacteroidetes* and *Firmicutes*^8^. Saunders *et al*. monitored the AS bacterial community compositions of 13 Danish wastewater treatment plants for 1 year. They determined that the genus *Nitrotoga* was the most abundant putative nitrite oxidizer^9^. All of these studies that quantified bacterial community structures in different WWTPs considered aeration tank samples to be representative of the whole WWTP^7,9–12^. However, the microbial communities in AS can be influenced by many possible factors, including the composition of influent, dissolved oxygen, treatment process, geographical location, and season^8–10,13,14^. Empirical tests for supporting this positive association between aeration tank AS and the AS of other biological treatment units, such as those of anoxic or anaerobic tanks, are lacking or are not fully supported by statistical analyses^15^. A few studies have considered temporal dynamics where long-term studies of the bacterial communities in activated sludge have reported temporal variations in both composition and structure^16–18^. However, these studies employed traditional molecular analysis methods such as automated ribosomal intergenic spacer region analysis (ARISA) or Terminal Restriction Fragment Length Polymorphism (T-RFLP) which yield results of fairly low accuracies due to PCR bias or low throughput^19^. Such low accuracy might have limited their conclusions about microbial community dynamics. In addition, most research on the AS microbial community of WWTPs has only focused upon one single system, usually represented as a municipal sewage treatment system^7,8,10,16–18,20–28^. Industrial wastewater treatment is a more difficult issue than domestic sewage where reaching discharge standards is often problematic due to the refractory organic pollutant level of the influent. A comprehensive comparison of domestic sewage treatment systems and industrial wastewater treatment systems would contribute valuable information for wastewater treatment practitioners towards improved design and maintenance of engineered industrial wastewater treatment systems.

In this year-round study, AS bacterial community and their seasonal succession of four typical WWTPs were represented. The core microbes in different wastewater treatment systems were then identified.

## Results

### Running condition of WWTPs

To maintain a relatively steady influent quality, all of the studied WWTPs had set up a regulating reservoir where the influent is pooled prior to entering the biological treatment facility. With the exception of industrial inflow rate, which decreased during the beginning of February 2016 (relating to the Chinese New Year vacation), these influent characteristics were maintained at a relatively stable state for all four plants. All plants operated stably whilst performing carbon and nitrogen removal. QG-A2O was also subjected to an enhanced biological phosphorus removal process^20,22,29^. In winter, their auxiliary heating system would maintain the biological treatment units at temperatures not lower than 13 °C^30^. The influent characteristics and purification performances of the 4 WWTPs are described as below (see also supporting material 1).

QG-A2O is a domestic sewage treatment plant which has adopted an A2/O process. Due to the removal of T-N and T-P being limited by the lack of carbon source, the system adds sodium acetate as additional carbon source^29^. Its incoming sewage had a better biodegradability (B/C is above 0.32) than other three WWTPs (the B/C being lower than 0.3). The effluent characteristics of QG WWTP was also significantly better than other two industrial wastewater treatment systems and was superior to the highest Chinese discharge 1-A standard^31^.

Compare with domestic sewage treatment system, the industrial wastewater treatment systems (SX-AO, SX-OD and SY-AO WWTP) display poor biodegradability. It should be ascribed to the abundance of refractory aromatic organic pollutants such as Benzene, Naphthalene, Anthracene, Quinone and their derivatives^32^. At the same time, their nitrogen and phosphate resources were relatively lower than those of the domestic wastewater treatment system^33^. In summer, due to the wastewater from the industrial enterprise bearing a certain amount of heat, the influent temperature could reach up to 40 °C. In this case the whole treatment efficiency was slightly declined and there was a need to increase the air supply to meet the microbial aeration requirement. The principal pollution treatment objectives for these plants were Chemical Oxygen Demand (COD) and chromaticity.

In the textile dyeing waste water treatment system (SX-AO and SX-OD WWTP). The influent contains high-concentrations of wastewater from textile dyeing, chemical, wine, food and pharmaceutical industries combined with municipal sewage from Shaoxing city. The largest influent proportion comes from textile-dyeing wastewater (about 80%). SX-AO adopted an A/O process whereas SX-OD adopted for an oxidation ditch process. The maximum influent COD could reach 1000 mg/L with a BOD range from 300 to 400 mg/L, with other characteristics of alkalinity and high chromaticity and with the wastewater containing contain dyestuff, sizing agent, oil, fiber and textile auxiliaries. Particular pollutants included terephthalic acid, ethylene glycol and some sulfides. The overall influent amount fluctuated with manufacturing levels between slack and busy seasons.

In the fine chemical wastewater treatment system (SY-AO WWTP), which mainly treating wastewater from synthetic dyestuff industry, the medical and bio-chemical industry together with some domestic wastewater. The WWTP’s influent also displays high chromaticity and has more complicated influent constituents than those of SX-AO and SX-OD, containing plenty of organic pollutants such as Benzene, Naphthalene, Anthracene, Quinone and their derivatives.

**Figure 1:**
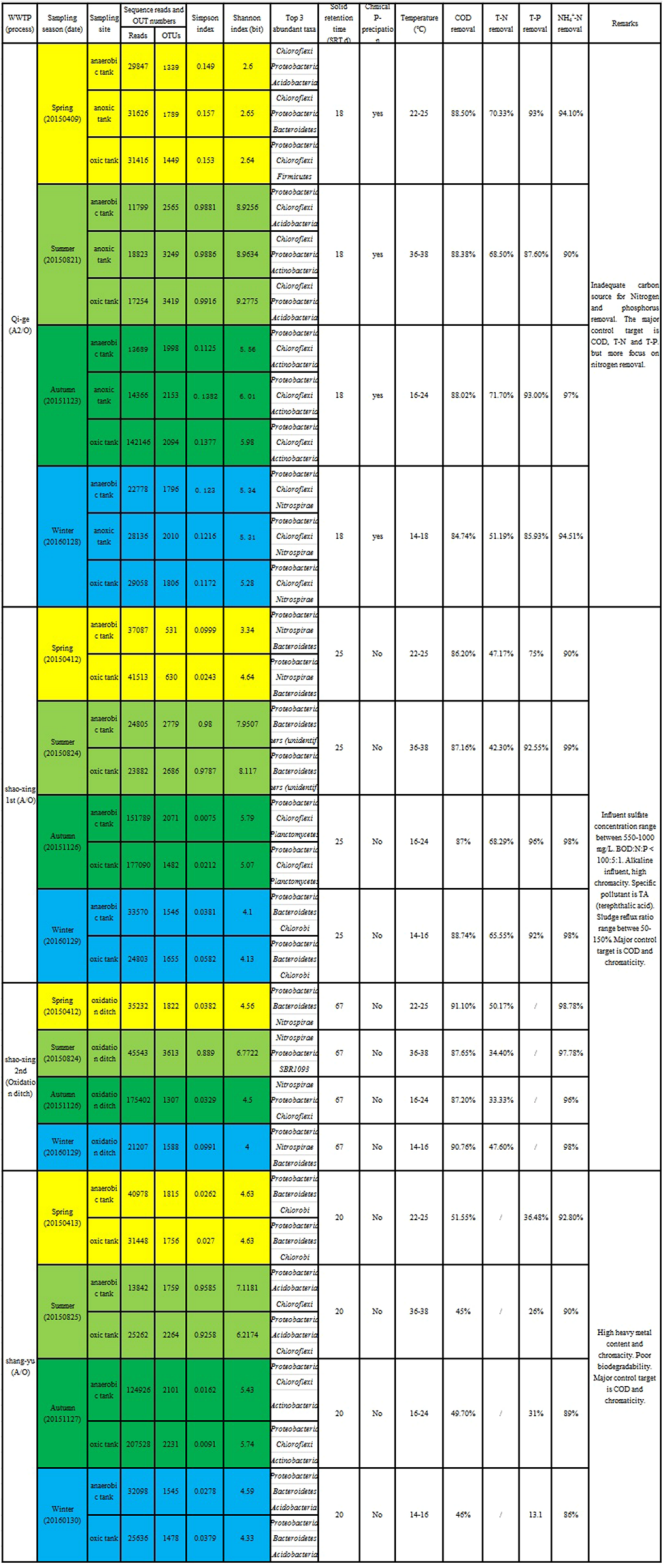
Summary of Illumina sequencing, microbial diversity, characteristics and average operational data of WWTP. OUT: Operational taxonomic unit, SRT: Solid retention time (**d**). COD removal: average monthly COD removal efficiency. T-N removal: average monthly total nitrogen removal efficiency. T-P removal: average monthly total phosphorus removal efficiency. NH_4_^+^-N removal: average monthly ammonia removal efficiency.

The overall performance differences of all the 4 WWTPs are summarized below. Specific performance data is shown in Fig. 1.

1. All WWTPs displayed comparatively stable COD removal rates which showed little seasonal fluctuation. The COD removal rate of SY-AO WWTP was the lowest represented (with an average of about 80%). The COD removal efficiency of QG-A2O WWTP did decreased a little in winter. The highest COD removal rate was achieved in SX-OD in spring (91.1%).
2. The T-N removal rate for these WWTPs was not as stable as the COD removal rates. These changed with season. The municipal sewage treatment system (QG-A2O) had a relative higher T-N removal rate (>68%). For the textile dyeing treatment system, the T-N removal rate of the oxidation ditch process (which was an average of 41.375% in SX-OD) was lower than that of the A/O process (with an average of 55.81% in SX-AO).
3. A highly stable NH_4_^+^-N removal rate was demonstrated in all WWTPs.
4. QG-A2O had adopted a chemical-aid phosphorus removal process. Therefore, its major control targets were those of COD and T-N. The major control target of the other 3 industrial wastewater treatment systems were COD and chromacity. In addition, due to the presence of too much synthetic dye in the industrial wastewater treatment systems, the influent of SX-AO, SX-OD and SY-AO usually contained sulfide^33,34^.

Generally speaking, domestic sewage treatment system has better inflow and therefore better pollutant removal performance than industrial one. Whereas the inflow to industrial wastewaters treatment system usually contain many refractory organic or inorganic pollutants, and adds pressure to their normal operation. The specific the raw wastewater of different industrial WWTP make their activated sludge samples exhibited unique bacterial community composition^13^.

### Bacterial community variation within biological treatment systems

The AS bacterial communities were analyzed based on high throughput sequencing technology. In total, 1684579 high quality reads of the 16S rRNA gene were obtained. The diversity indices are listed in Fig. 1. The average Shannon index of QG-A2O was 6.427 which was higher than others (5.45675, 4.956843, 5.228757 in SX-AO, SX-OD and SY-AO, respectively). Within the same WWTP, the Shannon index of the summer samples was higher than other 3 season’s samples. The top 3 taxa (at the phylum level) from the 32 samples are also summarized. As the Shannon index of the summer samples was higher than other seasons. Therefore, the summer sample comparisons were used to determine the variation among the different biological treatment units.

**Figure 2:**
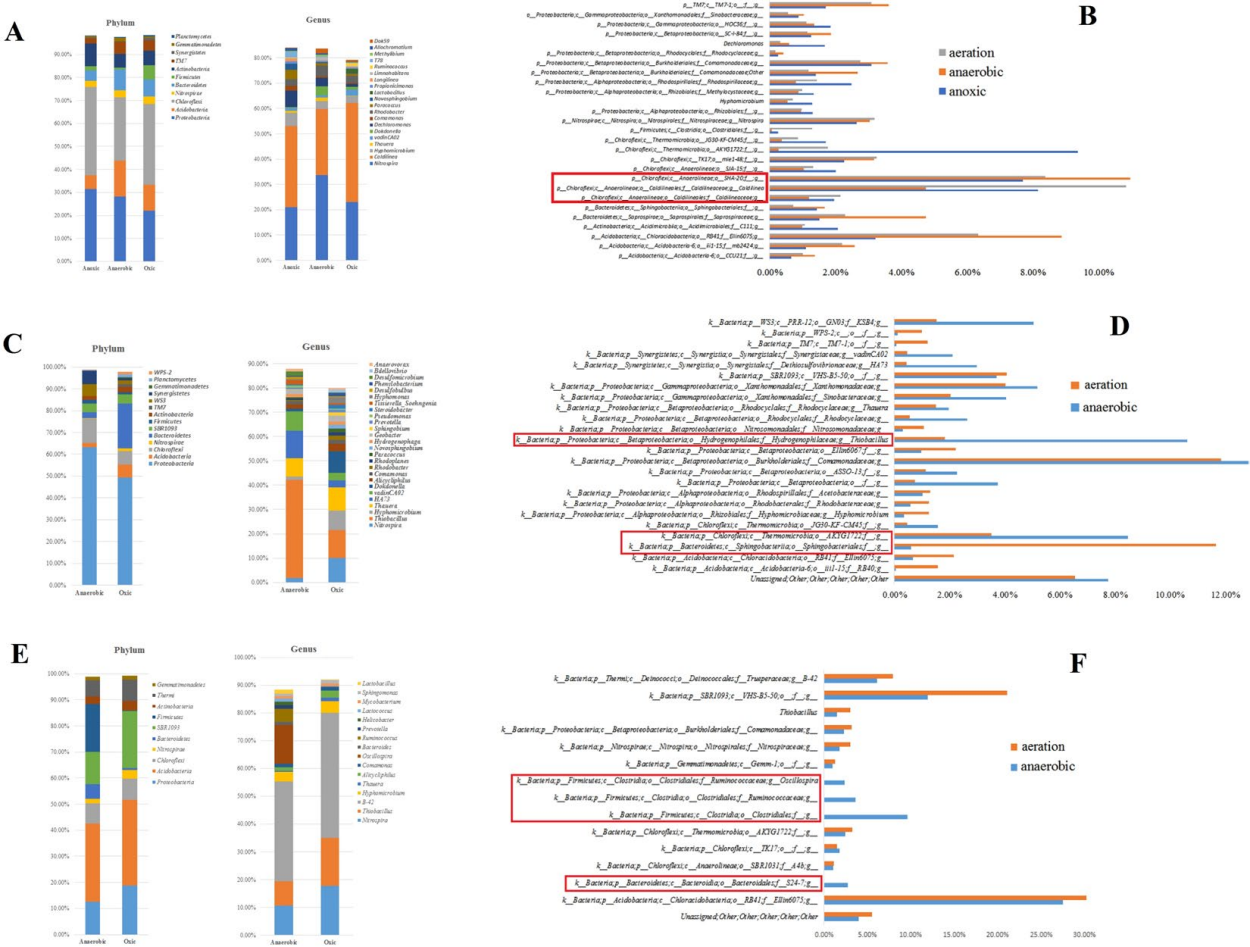
Comparison of bacterial community structure of different biological treatment units within one full-scale wastewater treatment system. Upper: Qc-A2/0 Middle: SX-AO Lower: SY-AO.

To investigate the variation among biological treatment units of A2/O processes, samples from the anoxic, anaerobic and aeration tanks of the QG-A2O WWTP were analyzed. The composition of the bacterial community in different biological treatment units (the anoxic, anaerobic and aeration tanks) was quite similar from phylum to genus level (Fig. 2A and supporting material 2). Although the members of *Anaerolineae* have been described as strictly anaerobic^35,36^, they were still found as abundant in every biological treat unit of the QG-A2O WWTP (Fig. 2B).

To better illustrate the bacterial community structure diversity within A/O process systems, the anaerobic and aeration tank samples of SX-AO and SY-AO WWTP were analyzed respectively because of their different inflow characteristic. In the A/O treatment system of textile dyeing wastewater (SX-AO WWTP), the AS bacterial community structure of anaerobic and aeration tanks were almost identical at the phylum level. Half of the bacterial community were *Proteobacteria*, followed by *Chloroflexi* and *Bacteroidetes*. *Bacteroidetes* was more abundant in the aeration tank (20.39% in the aeration tank whilst only 2.67% in the anaerobic tank). These bacterial community structural differences became more marked when probing on genus level (Fig. 2C and supporting material 2). The most distinctive and abundant microbe in the anaerobic tank was *Thiobacillus* (Fig. 2D).

In the A/O treatment system of fine chemical waste water (SY-AO WWTP), 10 kinds of bacterial phyla showed a relative abundance greater than 1% and were considered as high-ranking groups. Half of the bacteria were represented by just three groups (*Proteobacteria, Acidobacteria* and *SBR1093*). The bacterial community structure of the different tanks was quite similar with each other at the phylum level (Fig. 2E). However, on the genus level, *Oscillospira* and 3 unknown genera from *Bacteroidales* were much more abundant under anaerobic conditions (Fig. 2F and supporting material 2).

**Figure 3:**
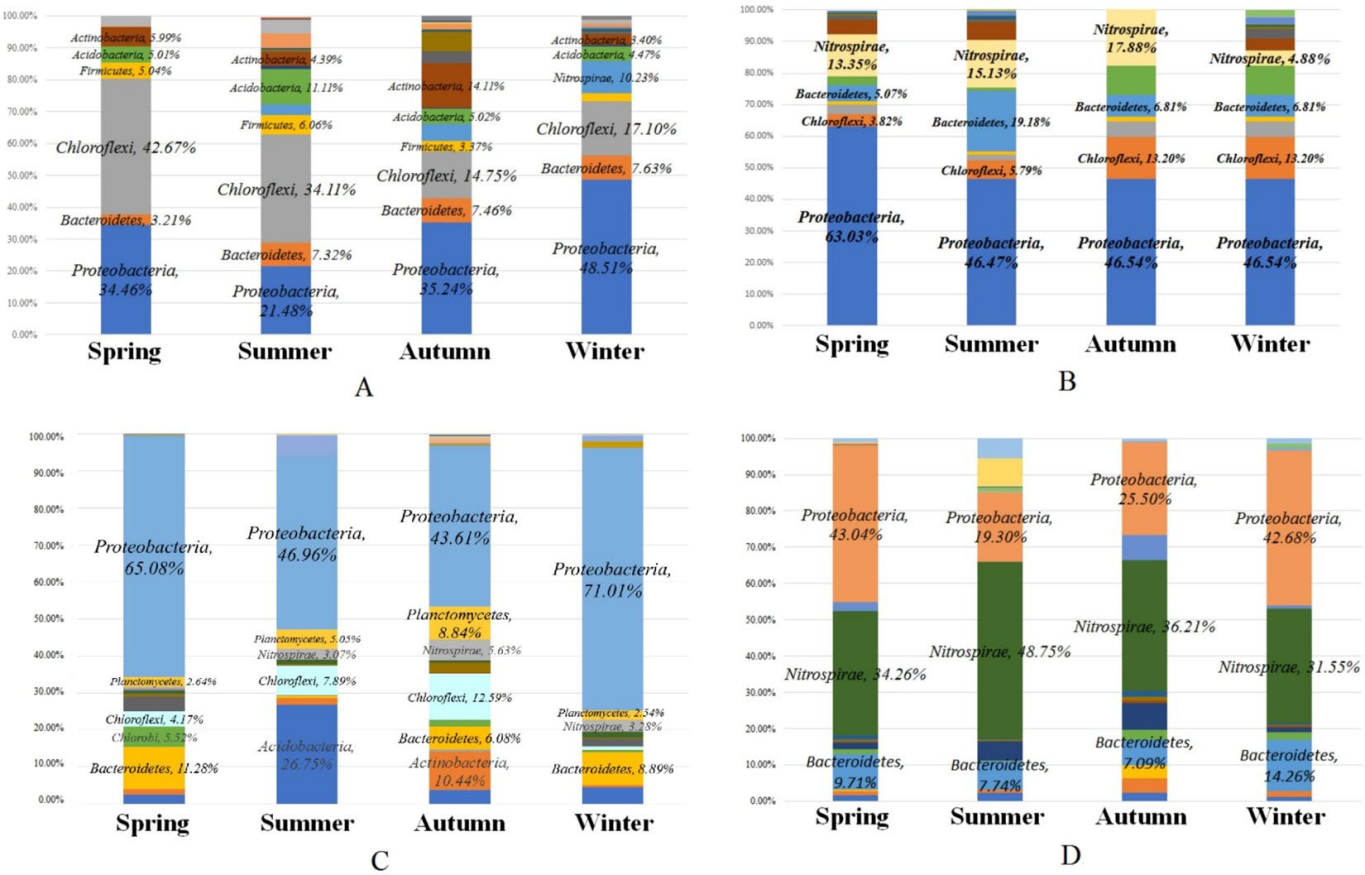
Bacterial community composition seasonal variation at phylum level. (**A**) Variation of QG-A2O. (**B**) Variation of SX-AO. (**C**) Variation of SY-AO. (**D**) Variation of SX-OD.

**Figure 4:**
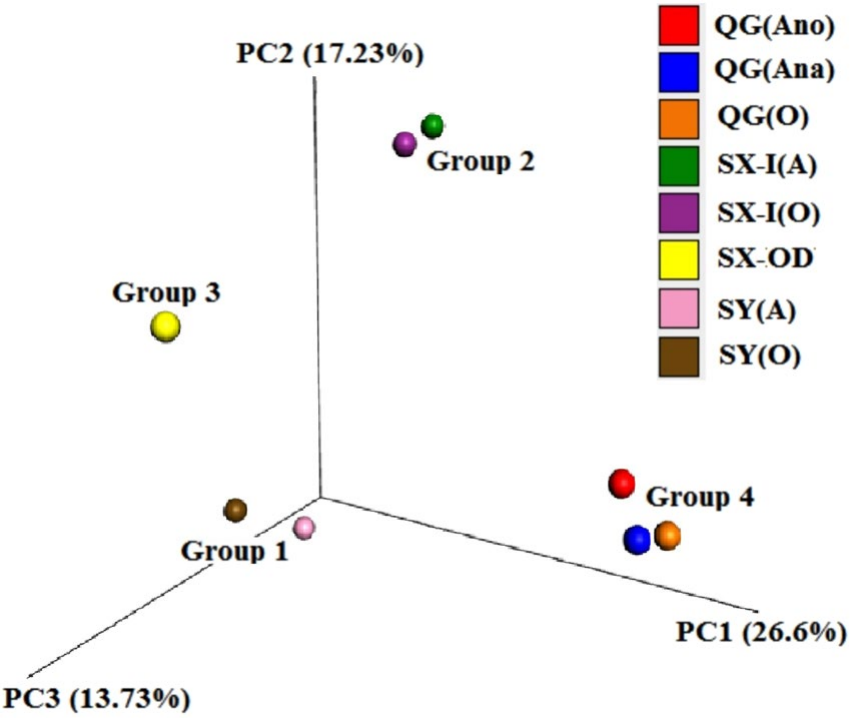
Principal coordinate analysis (PCoA) of summer samples.

As seen in Figs 2, 3 and 4, bacterial community of aeration tank, anaerobic tank and/or anoxic tank of A2/O or A/O processes displayed a similar microbial community structure, especially at the phylum level. Thus, despite some aspects microbial differences, the dominant microbes of different biological treatment units within A2/O and A/O systems is likely to be fairly similar. Based on this consistency, the taxonomic richness in the oxic tanks is adopted to illustrate seasonal effect on bacterial community.

### Bacterial community variation along with seasons

The effect of temporal variation on the AS bacterial community of A2/0 processes were investigated using the four seasonal samples of the QG-A2O WWTP. The impact of seasonal variation on the overall bacterial community composition can be seen in Fig. 3A. At the phylum level, the differences between the winter and autumn samples were less than the differences observed between summer and spring samples. With temperature increasing, the bacterial community became more complicated, where at a genera level, the bacterial community structure shifted significantly. In spring, the most predominant genera were *Lactococcus* (38.16%) and *Pseudomonas* (15.71%). In summer, the most predominant genus was *Caldilinea* (10.83%), followed by an unidentified genus from *Anaerolineae* (8.38%). In autumn, the most predominant genera were *Xanthomonadales-uncultured* (6.65%) and *Nitrospira* (5.19%). In winter, the most predominant genera were *Nitrospira* (10.23%) and *Caldilineaceae_uncultured* (6.87%).

The temporal variation effect on the AS bacterial community of A/O processes were investigated using the four seasonal samples, each taken from SX-AO and SY-AO WWTPs. In the A/O treatment system of textile dyeing wastewater (SX-AO), the impact of seasonal variation on the overall bacterial community composition can clearly be seen in Fig. 3B. At phylum level, the differences between the winter and autumn samples were less than the differences observed between summer and spring. Comparing to the spring sample, *Proteobacteria* had decreased significantly in the other three seasons. With the drop of air temperature (from summer to winter), *Chloroflexi* replaced *Bacteroidetes* became the secondary constituent which ranked only second to *Proteobacteria*. The bacterial community structure otherwise remained similar between these four season’s samples. At a genera level, the predominant genera in spring were *Nitrospira* (13.35%), *Thauera* (9.88%) and *Limnobacter* (6.23%) whereas the most predominant genera in summer were *Nitrospira* (15.43%), an unknown genus from *Comamonadaceae* (11.86%) and the order *Sphingobacteriales* (11.68%). Additionally, *Thauera* accounted for 1.5%. In autumn, the most predominant genera were *Nitrospira* (17.88), *Hyphomicrobium* (12.61%) and *Planctomyces* (5.76%). Additionally, *Thauera* accounted for 1.8%. In winter, the most predominant genera were *Thauera* (27.32%), an unknown genus from *Xanthomonadales* (10.16%) and *Nitrospira* (9.3%).

In the A/O treatment system of fine chemical engineering wastewater (SY-AO), despite some aspects microbial differences, the bacterial community structure essentially remained the same at the phylum level (Fig. 3C). The bacterial community structure essentially remained the same at the phylum level, though it is true that bacteria of the same phylum do not necessarily have identical metabolic functions. At the genera level, the most predominant genera in spring were *Thauera* (11.51%) and *Thiobacillus* (8.35%). The predominant genus in summer were unknown genes from the family *Ellin6075* (30.22%). In autumn, the most predominant genera were *Hyphomicrobium* (7.51%), *Planctomyces* (6.21%), *Nitrospira* (5.36%). In winter, the most predominant genera were *Thauera* (21.17%), *Thiobacillus* (10.57%), *Parvularcula* (5.97%) and *Nitrospira* (3.28%).

Due to the SX-OD WWTP adopting a carrousel oxidation ditch process, which is a kind of complete stirring activated sludge system. It has been reported that the anaerobic-, anoxic- and oxic-zones of the carrousel oxidation ditch share an approximately similar microbial community composition^20^. Therefore, the effect of temporal variation on bacterial community in the oxidation ditch was investigated only using the oxidation ditch sample of SX-OD WWTP. As shown in Fig. 3D, the dominant phylum showed almost no seasonal variation. Along with the rise of temperature, *proteobacteria* reduced from 42.68% to 19.3% and *Nitrospirae* increased from 31.58% to 48.75%. At a genera level, the dominant microbe-*Nitrospira* was stably greater than 31%.

## Discussion

The Shannon index can be used to measure microbial biodiversity, the higher value indicating the greater richness^37,38^. In this study, the highest average Shannon index was acquired in the QG-A2/O domestic WWTP (Fig. 1). That means that the phylogenetic diversity of the domestic sewage treatment systems was higher than the industrial ones. This same phenomenon has also been recorded in Argentina^13^.

The AS bacterial community in each biological treatment unit in the A2/O process system (QG-A2O) were quite similar from phylum level to genus level. For the two A/O process systems (SX-AO and SY-AO), the activated sludge bacterial communities of anaerobic and aeration tanks were also highly similar at the phylum level (Fig. 2C,E). However, at the genus level, the bacterial community structure in the anaerobic and oxic tanks did not differ in their dominant microbe compositions. This phenomenon could be ascribed to the high activated sludge reflux (external reflux) and/or aeration tank digestive fluid reflux (internal reflux) which had been conducted in A/O and A2/O process system. So, to some extent, it is reasonable to consider the oxic tank AS sample as representative of A/O and A2/O process systems. Compared with the high similarity of the AS bacterial community composition between anaerobic, anoxic and oxic tanks, known performance differences in these biological treatment units, such as nitrification and denitrification, might be caused by functional gene expression which is introduced by environmental parameters. To further illustrate the metabolic mechanism of a certain types of engineered system, further studies using more advanced technology such as metagenomic or metatranscriptomics analysis, are to be recommended.

While some common groups were shared by all samples, differences between samples became marked when probing at a genus level. Here, the abundant or distinct microbes which were presented in WWTPs the whole year-round is defined as the core genera. It is these core genera that are likely to be key microbes involved in the carbon, nitrogen metabolisms.

The highest and the lowest Shannon index were acquired in the summer and spring sample of QG-A2O domestic WWTP respectively (Fig. 1). This indicated a big fluctuation in microbial community. Despite this fluctuation, the COD, N, P levels of QG-A2O’s effluent remained consistent. This can be considered to be a functional redundancy^39,40^, where if some of the core genera can be replaced with others having the same functional role in active sludge, there may be no need for a dominant genera to be present. Although it is hard to determine the core microbes in QG-A2O, *Caldilinea*, a genus of fairly low abundance in other WWTPs, was represented in the QG-A2O WWTP over the whole year. As a member of class *Anaerolineae*, it has been reported as a PAO (phosphorus accumulating organism)^41^ and consists of some filamentous species which contribute to the floc stabilization of activated sludge^42^. Although the members of *Anaerolineae* have been described as strictly anaerobic^35,36^, Chen *et al*.^22^ also found a high representation of *Anaerolineae* in the aerobic treatment unit of a WWTP which is located only 150 kilometers away from the QG-A2O WWTP of this study. Both Chen’s WWTP and QG-A2O adopt A2/O processes and treat domestic sewage. This suggests that the genera from *Anaerolineae* may be widely representative of municipal activated sludge where the anaerobic microenvironment in the flocs of an aerobic unit may provide an ideal habitat. Additionally, the well-known nitrification function genus, *Nitrospira* were also found in QG-A2O throughout the year. In contrast, *Lactococcus* and *Pseudomonas* only accounted for high abundances in the spring sample (31% and 15.7% respectively). *Lactococcus* is a facultative anaerobic heterotrophic microbe from *Firmicutes* which has been reported to be able to utilize carbonhydrate to produce lactic acid, acetic acid, and formic acid at ambient temperatures^43,44^. *Pseudomonas* is a known denitrifying bacteria^45^. These two genera have been found to be dominant in aerobic granular reactors with good bio-degradable synthetic influent consisting of glucose, peptone and beef extract, and similar temperatures of about 30 °C^46^. Considering its operating conditions in spring, it seems that domestic sewage and the spring temperature agrees well with the acidogenesis characteristic of *Lactococcus*. Therefore, the core microbes in the QG-A2O WWTP might be nitrogen removal genera like *Nitrospira, Caldilinea, Pseudomonas* and the fermentative function microbe- *Lactococcus*.

In SX-AO and SX-OD, the core genera were *Nitrospira* and *Thauera*. These two genera presented stable abundances all over the year in the anaerobic tanks, oxic tanks and oxidation ditches. A recent study has indicated that *Nitrospira* are the most abundant and ubiquitous genus across the biological zones of the geography of different oxidation ditch WWTPs^20^. Comparatively, the content of *Nitrospira* in SX-OD was much higher (averaging above 15%) than those of previous reports. *Thauera* are involved in aromatic compound degradation^47,48^ and denitrification conditions^49,50^. High concentrations of organic pollutants in textile dyeing wastewater provides an excess of growth substrates for *Thauera*. Furthermore, *Thiobacillus* could also be found in all the samples of SX-AO and SX-OD. Although it accounted for less than 0.4% in the summer sample of SX-OD and autumn sample of SX-AO, it still accounted for more than 1% of all the other samples up to a maximum of 13.8% in the spring sample of SX-OD.

The core microbe of SY-AO, is hard to pin down because the dominant genera of this WWTP exhibited a high seasonal variation. The thermophilic microorganisms from the phylum *Thermi* (genus *B-42* of *Trueperaceae*) represent high abundances in summer (45%), but disappeared in others seasons. *Nitrospira*, *Thiobacillus* and *Thauera* were also presented all over the year but their content fluctuated considerably with seasons. *Nitrospira* account the highest amount of 17.68% in summer but only account 0.44% in spring. *Thiobacillus* presented more than 8% in spring, summer and winter but lower than 0.03% in autumn. *Thauera* presented more than 11.2% in spring and winter but lower than 0.9% in autumn. The roles of *Thiobacillus* and *Thauera* in autumn seem to have been largely replaced by some of the nitrification/denitrification functional bacteria such as *Hyphomicrobium*^51,52^, *Planctomyces*^53^ and *Nitrospira*^54^. This phenomenon might be caused by drastic changes in influent. For industrial wastewater, a steady and consistent discharge is difficult to maintain. Unpredictable cases of emergencies or accidents, sudden inspections, busy and slack seasons of manufacture and other fluctuating aspects of underlying discharge all combine to make consistent amount and composition of industrial wastewater unlikely and increase the likelihood of high fluctuation in influent characteristic. Although equalization tanks have been set at the front of these WWTPs which can mitigate such fluctuation to a limited extent, larger influent fluctuations are still going to have an impact. Nevertheless, in the absence of a dominant genera change caused by unexpectedly influent fluctuation, the core genera in SY-AO should remain *Nitrospira*, *Thiobacillus*, *Thauera*. *Thiobacillus* only uses non-organic carbon sources such as CO_3_^2−^ and HCO_3_ and has been previously reported as a sulfur-based autotrophic denitrifier^55–57^. Since SY-AO mainly treats wastewater from pharmaceutical, synthetic dye and dyeing industry, the high sulfate and refractory organic pollutant concentration in the influent^58^ may contribute to the ecological amplitude of *Thiobacillus* and *Thauera*. These two microbes were also the dominant genera in WWTPs treating coking wastewater^23^. Their presence can be explained by their ability to biodegrade specific components of the industrial wastewater, like nitrobenzene, anthraquinone, indole etc^6,13^.

Taken together, *Nitrospira*, *Thauera* and *Thiobacillus* were the core genera in textile dyeing and fine chemical industrial wastewater treatment systems^6,13^.

The PCoA plot (Fig. 4) indicates that the bacterial community differences within a full-scale wastewater treatment system is much lower than the differences between systems. For example, the two sludge samples of SX-AO (SX-anaerobic and SX-oxic) were displayed similarities, but differed considerably to the samples of the SY-AO and QG-A2O WWTPs (supporting material 3). In this, the influent nature and sources of the three (Shaoxing, Qige and Shangyu) WWTPs should be distinct. There was a particular contrast between the influent characteristics of SX-AO and SY-AO despite them adopting the same A/O process. With this it is the different influent of SX-AO and SY-AO that made their bacterial community composition vary and cause these two to be clustered into different groups according to the PCoA plot. So, influent type is the primary determinant of the unique composition of WWTP bacterial communities. However, although dealing with the same influent type, the results from samples SX-OD did not correspond well with those of SX-AO. This indicates that process is also an important determinant of the bacterial community composition. Furthermore, although the samples of different biological treatment units within a wastewater treatment system were distinctly grouped together with the lowest between-sample variation on the PCoA plot due to their similar bacterial community composition, their differences in microbial community composition still can be observed at the genera level (Fig. 2B,D,F). This may be ascribed to the differences in dissolved oxygen concentrations. In this way, the dissolved oxygen concentration is another important determinant of a unique bacterial community. The idea that process and dissolved oxygen are the prime influencers of community structure of biological treatment systems has been suggested previously^14,17^. However, previous studies had concluded that dissolved oxygen concentration could not be used to group samples into meaningful categories^13^. In this study, according to the result of PCoA, the effect that process has on microbial community composition is essentially equal to the effect of dissolved oxygen. As the nature of process has a direct relationship upon dissolved oxygen, therefore either process or dissolved oxygen concentration as the secondary factor determining the ultimate sample classification.

In another aspect, the fact that the highest Shannon index was gained in the summer samples and that there was a wide difference in Shannon index of four seasons indicates that seasonal variation sends shock-waves into the bacterial community where increasing temperature dramatically improves the microbial diversity. Facing such seasonal temperature fluctuation shock, the oxidation ditch process seems to be more stable than the A2/O and A/O processes. For the OD, the dominant genera-*Nitrospira* were maintained stably and at a high percentage (31.2–48.68%). These data suggested a slow community turnover within plants and a high impact of the seasonal temperature change on the bacterial community composition.

**Figure 5:**
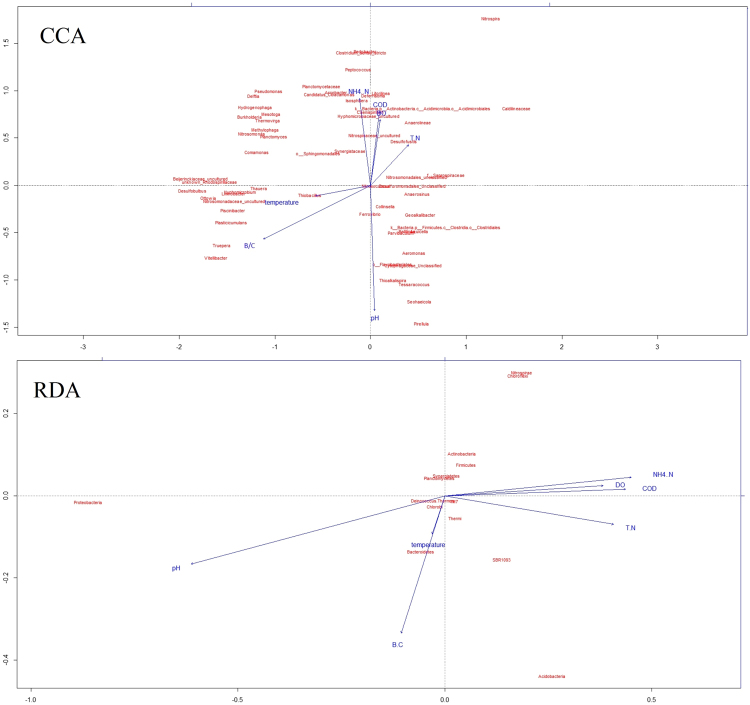
CCA and RDA analysis plot. The arrow length represents the strength of the correlation between the environmental variables and the microbes. The longer the arrow length, the stronger the correlation. The perpendicular distance between microbes and environmental variable axes in the plot reflects their correlations. The smaller the distance, the stronger the correlation. B/C = BOD_5_/COD_Cr_. pH = Influent pH value. NH4 + -N = ammonia nitrogen removal efficiency. TN = total nitrogen removal efficiency. COD = Chemical Oxygen Demand removal efficiency. DO = dissolved oxygen. Temperature: influent temperature.

To visualize the relationship between microbe and environment/operation parameters of the processes in the WWTPs, Canonical Correspondent Analysis (CCA) and Redundancy Analysis (RDA) were performed at the genus and phylum level respectively (Fig. 5). *Thauera*, *Thiobacillus*, *Truepera*, *Vitellibacter*, *Nitrosomonadaceae* were positively correlated with temperature and better influent biodegradability (B/C value) while negatively correlated with T-N removal rate. In contrast, *Nitrospira*, A*naerolinea*, D*esulfofustis*, C*aldilineaceae* were positively correlated with the T-N removal rate while they were negatively correlated with better influent biodegradability. *Planctomycetaceae*, *Arcobacter*, *Isosphaera*, *hyphomicrobiaceae*, *Nitrospinaceae peptoccoccus* were positively correlated with NH4-N removal rate. The seasonal effect, primarily represented by temperature, on the bacterial community structure of WWTPs has been noted previously where some have concluded that temperature is the dominant environmental variable affecting the bacterial community^16^. However, in this study, the temperature effect was not so remarkable factor as influent biodegradability, dissolved oxygen and pH value. At a genus level (Fig. 5-CCA analysis), the abundance of *Thiobacillus*, *Thauera* and *Nitrosomonadaceae* presented strong positive correlations with temperature. Xu also reports that the abundance of *Thauera* had a positive correlation with temperature^20^. At the phylum level (Fig. 5-RDA analysis), previous studies found that *Saprospiraceae* and *Alphaproteobacteria* were temperature-sensitive in an A2/O system^18^. In contrast, *Proteobacteria* was not sensitive to temperature, whilst *Thermi*, *chlorobi* and *Bacteroidetes* did present positive correlations with temperature. Such observational differences might be ascribed to differing microbial community analytic technology.

## Conclusion

Bacterial communities in anaerobic tanks, anoxic tanks and aeration tanks of A2/O process systems all display very similar microbial community structures which are also broadly similar to the bacterial communities of A/O process system. AS sample differences within WWTPs was lower than the differences between WWTPs. Comparatively, seasonal variation has a fairly strong impact on the bacterial community. However, this factor was inferior to the effect of influent type and type of process. Compared with A2/O and A/O processes systems, the microbial community in the oxidation ditch process was more stable between different seasons. The sludge from domestic sewage treatment had greater diversity than the sludge from industrial wastewater treatment. The core genera in domestic wastewater treatment systems were *Nitrospira*, *Caldilinea*, *Pseudomonas* and the fermentative function microbe-*Lactococcus*. Whereas, *Nitrospira*, *Thauera* and *Thiobacillus* were highly involved in the textile dyeing and fine chemical industrial wastewater treatment system.

## Material and Methods

### Samples collection

We investigated 4 full-scale wastewater treatment systems belonging to the WWTPs of three cities located in Zhejiang province, eastern China, namely Shaoxing (SX), Qige (QG) and Shangyu (SY). Samples taken from these WWTPs incorporated Anaerobic-Oxic (A/O), Anaerobic-Anoxic-Oxic (A2/0) and/or Oxidation Ditch (OD) processes. The samples are hereafter represented as SX-AO, SX-OD, QG-A2O and SY-AO. During stable operating circumstances, we sampled 200 mL wastewater containing suspended biomass from the front end, middle and combined outlet (600 ml total) for each biological treatment unit. These samples were then combined and mixed together. To consider temporal variations, samples were obtained from four separate seasons at the same locations between March 2015 and April 2016. The samples were numbered chronologically and stored at −80 °C before use. The descriptions of these samples are shown in Fig. 1. Influent and effluent quality data from the routine process monitoring was obtained using standard analytical procedures^59^, which were collected from online databases of local environmental protection agencies and WWTPs.

### DNA extraction, PCR amplification and Illumina sequencing

Biomass was collected via the centrifugation of samples at 4,000 rpm for 5 min at room temperature. The supernatants were then decanted. Samples were then pretreated using the liquid nitrogen grinding method before using a DNA Isolation Kit (E.Z.N.A., Omega, Norcross, Georgia, U.S.) to extract sample DNA according the manufacturer’s instructions. The purity and the quantity of extracted DNA was determined by UV spectrophotometry at 260 and 280 nm. DNA extracts were stored at −20 °C.

Bacterial 16S rRNA gene fragments were PCR-amplified with the primers. 340F (5′-CCTACGGGNBGCASCAG -3′) and 806 R (5′-GGACTACNVGGGTATCTAAT-3′). The primer set could amplify fragments corresponding to the V3-V4 hypervariable regions of the 16S rRNA. PCR reactions were performed in triplicate with a 20 μl of mixture containing 4ul of 5 × FastPfu buffer. All PCR amplifications were performed using an ABI GeneAmp® 9700 thermocycler. Each PCR reaction was performed in triplicate with 20 μL of mixture containing 4 μL of 5 × FastPfu Buffer, 2 μL of 2.5 mM dNTPs and 0.8 μL of each primer (5 μM). Amplicons were extracted from 2% agarose gels and purified using an AxyPrep DNA Gel Extraction Kit (Axygen Biosciences, Union City, CA, U.S.) according to the manufacturer’s instructions and quantified using QuantiFluor™-ST (Promega, U.S.).

Purified amplicons were pooled in equimolar amounts and paired-end sequenced (2 × 250) on an Illumina MiSeq platform according to the standard protocols. The sequences were then deposited in the NCBI Short Read Archive under accession number: SRP110572.

### High-throughput sequencing data analysis

Raw fastq files were demultiplexed, quality-filtered by Trimmomatic and merged by FLASH according to the following criteria: (i) The 250 bp reads were truncated at any site receiving an average quality score of <20 over a 10 bp sliding window. Truncated reads shorter than 50 bp were discarded. (ii) Exact barcode matching: Sequences with 2 nucleotide mismatches in primer matching, or reads contains ambiguous characters were removed. (iii) Only sequences which overlapped for more than 10 bp were assembled according to their overlap sequence. Reads which could not be assembled were discarded.

Operational Taxonomic Units (OTUs) were clustered with a 97% similarity cutoff using UPARSE (version 7.1 http://drive5.com/uparse/) and chimeric sequences were identified and removed using UCHIME. The phylogenetic affiliation of each 16S rRNA gene sequence was analyzed using a RDP Classifier algorithm (http://rdp.cme.msu.edu/) against the Silva (SSU123) 16S rRNA database using a confidence threshold of 70%. Species diversity, richness was computed at 3% dissimilarity as part of alpha diversity pipeline.

Principal-Coordinate Analysis (PCoA), Canonical Correspondence Analysis (CCA) and Redundancy Analysis (RDA) were performed in the R environment with the vegen package^60^. PCoA was used to group the microbial communities of different samples. CCA and RDA were used to investigate the relationship of the measured variables as well as their impacts upon the performance of the wastewater treatment processes.

## Electronic supplementary material

supporting material files
