# Supplementary material for: Seasonal bacterial community succession in four typical wastewater treatment plants: correlations between core microbes and process performance
Source: Sci Rep. 2018 Mar 15;8:4566. doi: 10.1038/s41598-018-22683-1 (PMC5854665; doi:10.1038/s41598-018-22683-1)

Seasonal bacterial community succession in 4 typical wastewater treatment plants: correlations between core microbes and process performance

Bo Zhang[a], Quanwei Yu[b], Guoqi Yan[c], Hubo Zhu[d], Xiang yang Xu[a,b], Liang Zhu*[a,b]

a. Department of Environmental Engineering, Zhejiang University, Hangzhou 310058, China

b. Zhejiang Province Key Laboratory for Water Pollution Control and Environmental Safety, Hangzhou 310058, China

c. Hangzhou Drainage Co., LTD, East City Water Treatment Branch, Hangzhou 310018, China

d. Shangyu Wastewater Treatment Plant, Shangyu 312369, China

*Corresponding author: Liang Zhu

Address: Department of Environmental Engineering, Zhejiang University, No. 866 Yuhangtang Road, Hangzhou 310058, China.

Tel.: +8657188982343; Fax: +86 571 88982343

E-mail address: felix79cn@hotmail.com

Supporting material 1

Table S1: COD value of influent and effluent (mg/liter)

Date	QG-A2O		SX-AO		SX-OD		SY-AO	
	Infuent monthly average	effluent monthly average	Infuent monthly average	effluent monthly average	Infuent monthly average	effluent monthly average	Infuent monthly average	effluent monthly average
January-15	337.28	22.56	437	97	672	92.3	293.64	170
Februaty-15	228.06	23.77	386	94.8	1561	91.6	329.00	169.00
March-15	199.54	19	486	93.4	691	97	355.00	117.00
April-15	277.71	27.42	587	105	1372	109	322.00	156.00
May-15	261.5	19.23	520	111	1419	102	306.00	145.00
June-15	192.03	18.71	448	95.8	482	89.2	274.00	148.00
July-15	217.67	15.4	378	91.1	512	83.3	260.00	137.00
August-15	246	21.26	446	95.1	386	95.1	278.00	152.00
September-15	292.48	21.97	472	101	448	109	294.00	147.00
October-15	297	23.83	457	96.4	465	85.3	299.00	158.00
November-15	265.23	22.32	401	87.2	557	76.1	336.00	169.00
December-15	262.33	25.61	538	90.1	658	84.3	377.00	188.00
January-16	186.36	26.44	493	85.7	855	78.3	465.00	251.00
February-16	176.26	19.48	443	83.1	290	68.1	439.00	187.00
March-16	332.17	31.59	639	92.3	638	83.8	396.00	229.00
April-16	328.71	24.97	692	74.9	493	85.4	369.00	211.00

Table S2: Ammonia nitrogen value of influent and effluent (mg/liter)

Date	QG-A2O		SX-AO		SX-OD		SY-AO	
	Infuent monthly average	effluent monthly average	Infuent monthly average	effluent monthly average	Infuent monthly average	effluent monthly average	Infuent monthly average	effluent monthly average
January-15	32.46	2.692	59.7	0.684	65.3	0.588	38.89285714	5.75
February-15	24.41	0.455	43.8	0.661	53.6	3.79	40	3.24
March-15	21	0.4	45.9	1.51	55.7	18.1	31.4	9.22
April-15	24.9	0.6	65	2.55	72.8	1.04	34	2.45
May-15	25.58	0.53	71.1	1.56	74.7	0.926	33.5	2.31
June-15	19.7	0.27	58	1.43	65.4	1.3	28.6	2.06
July-15	19.37	0.6	52.4	1.18	57.3	3.67	23.2	2.28
August-15	21.2	0.358	54.9	0.933	57.3	1.94	27	2.67
September-15	25.3	0.197	49.9	1.05	59.2	1.43	24.7	2.14
October-15	25.73	0.16	52.2	1.51	65.7	1.43	22	2.14
November-15	26.76	0.458	51	0.758	58.7	1.12	18.6	1.96
December-15	28.6	0.83	66.2	8.91	66.9	1.08	21.2	2.89
January-16	21.3	0.3148	61.3	1.25	72.5	1.87	29.7	26.9
February-16	20.78	1.058	57.7	14.4	57.1	25.4	45.4	39.2
March-16	32.36	2.66	99.2	8.97	93.4	2.28	29.7	28.7
April-16	30.2	1.2	85.1	2.73	102	1.45	35.7	16.3

Table S3: Total nitrogen value of influent and effluent (mg/liter)

Date	QG-A2O		SX-AO		SX-OD		SY-AO	
	Infuent monthly average	effluent monthly average	Infuent monthly average	effluent monthly average	Infuent monthly average	effluent monthly average	Infuent monthly average	effluent monthly average
January-15	40.48	11.44	77.5	26.7	75.8	50.9	/	/
February-15	31.35	10.32	53.9	49	75.2	53.3	/	/
March-15	26.86	10.61	63.1	44.2	75.2	37.6	/	/
April-15	31.81	9.87	78.8	49.7	82.8	51.9	/	/
May-15	33.10	9.57	81.5	55.6	92.3	52.9	/	/
June-15	24.81	8.77	74.1	42.7	73.6	48.3	/	/
July-15	25.23	8.97	65.5	37.2	67.8	37.7	/	/
August-15	26.42	8.32	68.8	41	70.4	43.5	/	/
September-15	31.74	9.23	63.5	43.6	69.8	26.4	/	/
October-15	33.27	10.70	65.6	45.4	78.7	42.8	/	/
November-15	32.29	10.29	65.1	36.6	73.4	38.5	/	/
December-15	34.31	11.00	87.3	42.7	87.6	40.1	/	/
January-16	26.56	10.76	67.6	48.5	86.5	53.7	/	/
February-16	24.42	10.55	57.7	/	64.1	49	/	/
March-16	37.79	11.21	126	65.2	112	50.8	/	/
April-16	37.10	11.35	106	56	118	58.8	/	/

Table S4: Total phosphate content of influent and effluent (mg/liter)

Date	QG-A2O		SX-AO		SX-OD		SY-AO	
	Infuent monthly average	effluent monthly average	Infuent monthly average	effluent monthly average	Infuent monthly average	effluent monthly average	Infuent monthly average	effluent monthly average
January-15	2.80	0.26	6.74	0.145	5.26	0.046	7.47	6.38
February-15	2.46	0.28	4.99	0.481	4.51	0.36	7.01	5.95
March-15	2.20	0.30	5.2	0.465	4.58	0.294	8.66	4.80
April-15	2.51	0.32	3.36	0.28	3.06	0.204	7.21	4.58
May-15	2.71	0.23	4.16	0.343	3.61	0.051	6.22	4.50
June-15	2.39	0.28	4.78	0.242	4.51	0.104	6.50	5.20
July-15	2.32	0.28	3.35	0.191	3.56	0.176	6.45	5.23
August-15	2.42	0.31	2.76	0.254	2.93	0.107	8.41	6.21
September-15	2.82	0.35	2.92	0.227	2.9	0.114	14.70	12.70
October-15	2.88	0.35	3.34	0.072	2.7	0.033	15.00	15.70
November-15	2.64	0.30	2.6	0.091	3.06	0.066	17.40	12.00
December-15	2.54	0.41	3.8	0.1	4.12	0.07	16.10	15.30
January-16	2.20	0.35	4.07	0.158	5.13	0.039	20.60	17.90
February-16	2.12	0.36	9.12	/	4.14	0.06	19.90	10.00
March-16	2.78	0.45	4.23	0.321	5.5	0.119	15.70	13.00
April-16	2.79	0.26	4.66	0.127	3.73	0.353	10.90	10.00

Supporting material 2

Table S5: Activated sludge bacterial community composition at the phylum level (%)

TaxonName	QG(Ano)	QG(Ana)	QG(O)	SX-1-(A)	SX-1-(O)	SX.2.OD	SY(A)	SY(O)
Proteobacter	31.50%	28.23%	22.01%	63.19%	49.42%	20.33%	12.63%	18.68%
Acidobacteri	5.99%	15.51%	11.38%	1.93%	5.93%	2.45%	29.96%	33.00%
Chloroflexi	38.32%	27.56%	35.04%	11.23%	6.19%	5.60%	7.71%	8.20%
Nitrospirae	2.69%	3.13%	3.28%	0.31%	1.20%	51.36%	1.83%	3.19%
Bacteroidete:	4.53%	8.96%	7.50%	2.67%	20.39%	8.15%	5.61%	0.78%
SBR1093	0.16%	0.24%	0.12%	4.01%	4.33%	8.18%	12.27%	21.93%
Firmicutes	1.91%	0.90%	6.20%	1.71%	1.08%	0.39%	18.36%	0.19%
Actinobacter	9.75%	5.93%	6.28%	1.57%	2.34%	1.17%	2.90%	3.53%
Thermi	0.03%	0.14%	0.04%	0.04%	0.06%	0.20%	6.26%	8.20%
TM7	2.28%	5.35%	4.59%	0.09%	1.32%	1.35%	0.30%	0.31%
WS3	0.15%	0.39%	0.11%	5.44%	1.63%	0.02%	0.18%	0.08%
Synergistetes	0.48%	0.33%	1.05%	6.09%	1.04%	0.04%	0.03%	0.02%
Gemmatimor	0.54%	1.45%	0.62%	0.04%	0.53%	0.21%	1.27%	1.50%
Planctomycet	0.24%	0.41%	0.61%	0.41%	1.25%	0.05%	0.19%	0.05%
WPS-2	0.54%	0.74%	0.65%	0.13%	1.07%	0.01%	0.00%	0.02%
Chlorobi	0.46%	0.38%	0.19%	0.38%	0.55%	0.24%	0.12%	0.11%
Cyanobacteri	0.06%	0.04%	0.03%	0.10%	0.59%	0.08%	0.23%	0.10%
Verrucomicro	0.07%	0.05%	0.06%	0.17%	0.22%	0.00%	0.05%	0.01%
FBP	0.00%	0.00%	0.00%	0.07%	0.19%	0.01%	0.00%	0.00%
Spirochaetes	0.02%	0.04%	0.01%	0.35%	0.27%	0.00%	0.01%	0.02%
Armatimona	0.00%	0.02%	0.01%	0.02%	0.16%	0.08%	0.00%	0.00%
TM6	0.03%	0.05%	0.02%	0.02%	0.01%	0.03%	0.02%	0.04%
BRC1	0.00%	0.00%	0.00%	0.01%	0.12%	0.02%	0.00%	0.00%
OP8	0.03%	0.02%	0.10%	0.00%	0.00%	0.00%	0.00%	0.00%
Chlamydiae	0.02%	0.01%	0.02%	0.00%	0.00%	0.00%	0.05%	0.02%
WS6	0.06%	0.01%	0.03%	0.00%	0.00%	0.00%	0.02%	0.00%
OD1	0.04%	0.05%	0.01%	0.00%	0.00%	0.00%	0.00%	0.00%
NKB19	0.01%	0.00%	0.00%	0.00%	0.02%	0.00%	0.00%	0.02%
SR1	0.02%	0.03%	0.02%	0.00%	0.00%	0.00%	0.00%	0.00%
OC31	0.00%	0.00%	0.00%	0.00%	0.03%	0.00%	0.00%	0.00%
OP9	0.00%	0.00%	0.00%	0.01%	0.02%	0.00%	0.00%	0.00%
GN04	0.02%	0.04%	0.01%	0.00%	0.00%	0.00%	0.00%	0.00%
Elusimicrobia	0.02%	0.00%	0.00%	0.00%	0.00%	0.00%	0.00%	0.01%
Lentisphaera	0.00%	0.00%	0.00%	0.00%	0.02%	0.00%	0.00%	0.00%
WS2	0.02%	0.00%	0.00%	0.00%	0.00%	0.00%	0.00%	0.00%
GOUTA4	0.00%	0.00%	0.00%	0.00%	0.00%	0.00%	0.00%	0.00%
Fusobacteria	0.00%	0.01%	0.00%	0.00%	0.00%	0.00%	0.00%	0.00%
MVP-21	0.00%	0.00%	0.00%	0.00%	0.00%	0.00%	0.00%	0.00%
GN02	0.00%	0.01%	0.01%	0.00%	0.00%	0.00%	0.00%	0.00%

Table S6: Activated sludge bacterial community composition at the class level (%)

TaxonName	QG(Ano)	QG(Ana)	QG(O)	SX-1-(A)	SX-1-(O)	SX.2.OD	SY(A)	SY(O)
Betaproteobacteria	7.85%	10.38%	6.01%	38.06%	23.18%	7.35%	5.27%	8.83%
Chloracidobacteria	2.29%	7.73%	5.14%	0.66%	2.16%	0.67%	25.89%	28.64%
Nitrospira	3.71%	4.72%	4.68%	0.44%	1.55%	65.16%	1.69%	2.93%
Alphaproteobacteria	9.90%	6.22%	6.44%	5.75%	11.43%	4.44%	4.67%	6.50%
VHS-B5-50	0.11%	0.18%	0.09%	3.57%	4.04%	5.19%	11.28%	19.99%
Anaerolineae	16.79%	16.79%	19.76%	0.16%	0.50%	0.80%	1.06%	1.18%
Thermomicrobia	7.74%	0.52%	1.92%	9.67%	3.96%	1.56%	2.88%	3.62%
Gammaproteobacteria	3.46%	3.75%	2.68%	10.34%	9.80%	0.92%	0.85%	1.22%
Sphingobacteriia	1.01%	1.30%	0.52%	0.58%	11.59%	4.09%	0.02%	0.03%
Saprospirae	1.50%	4.44%	2.44%	0.25%	6.20%	0.74%	0.29%	0.41%
Clostridia	1.04%	0.61%	3.68%	1.28%	0.71%	0.21%	16.35%	0.13%
Acidobacteria-6	1.37%	3.53%	2.64%	0.16%	2.33%	0.78%	1.43%	1.17%
TK17	1.62%	2.73%	2.65%	0.13%	1.04%	0.80%	2.36%	2.00%
Deinococci	0.02%	0.10%	0.03%	0.03%	0.06%	0.13%	5.75%	7.48%
TM7-1	1.19%	2.80%	2.27%	0.06%	1.21%	0.82%	0.11%	0.11%
Acidimicrobiia	2.71%	2.10%	1.87%	0.79%	1.39%	0.34%	0.07%	0.14%
PRR-12	0.10%	0.29%	0.08%	4.84%	1.52%	0.01%	0.16%	0.07%
Actinobacteria	6.72%	4.47%	4.49%	1.40%	2.19%	0.74%	2.66%	3.22%
Bacteroidia	0.38%	0.29%	1.90%	1.46%	0.77%	0.19%	4.69%	0.06%
Synergistia	0.33%	0.25%	0.75%	5.42%	0.97%	0.03%	0.03%	0.02%

Deltaproteobacteria	0.47%	0.89%	0.48%	1.89%	1.57%	0.18%	0.48%	0.45%
Gemm-1	0.28%	0.70%	0.26%	0.04%	0.25%	0.09%	0.95%	1.18%
Flavobacteriia	0.21%	0.64%	0.48%	0.07%	0.32%	0.09%	0.14%	0.20%
Bacilli	0.28%	0.06%	0.62%	0.24%	0.30%	0.04%	0.42%	0.04%
S085	0.01%	0.07%	0.04%	0.01%	0.03%	0.11%	0.60%	0.57%
iii1-8	0.06%	0.13%	0.08%	0.66%	0.73%	0.00%	0.00%	0.01%
Ignavibacteria	0.13%	0.11%	0.06%	0.31%	0.38%	0.12%	0.11%	0.10%
Solibacteres	0.06%	0.15%	0.04%	0.23%	0.30%	0.08%	0.13%	0.25%
4C0d-2	0.02%	0.01%	0.01%	0.09%	0.50%	0.05%	0.19%	0.09%
Planctomycetia	0.10%	0.18%	0.37%	0.15%	0.42%	0.00%	0.06%	0.01%
TM7-3	0.22%	0.20%	0.28%	0.01%	0.02%	0.02%	0.16%	0.17%
OM190	0.05%	0.04%	0.04%	0.08%	0.53%	0.00%	0.04%	0.02%
SC3	0.07%	0.67%	0.47%	0.00%	0.00%	0.00%	0.00%	0.00%
Ellin6529	0.09%	0.12%	0.08%	0.02%	0.03%	0.08%	0.14%	0.06%
Sva0725	0.67%	0.28%	0.47%	0.00%	0.00%	0.04%	0.00%	0.00%
TA18	0.02%	0.04%	0.09%	0.01%	0.04%	0.02%	0.08%	0.04%
Gemmatimonadetes	0.37%	1.09%	0.45%	0.04%	0.50%	0.13%	1.16%	1.37%
TK10	0.04%	0.01%	0.05%	0.01%	0.15%	0.09%	0.04%	0.04%
Epsilonproteobacteria	0.00%	0.00%	0.05%	0.22%	0.07%	0.00%	0.26%	0.00%
Cytophagia	0.02%	0.08%	0.01%	0.01%	0.09%	0.06%	0.01%	0.01%
SJA-28	0.17%	0.06%	0.04%	0.00%	0.03%	0.03%	0.00%	0.00%
Phycisphaerae	0.01%	0.01%	0.00%	0.11%	0.03%	0.03%	0.06%	0.02%
Gitt-GS-136	0.00%	0.00%	0.00%	0.00%	0.00%	0.08%	0.00%	0.00%
Chloroflexi	26.40%	20.77%	25.07%	10.00%	5.78%	3.56%	7.08%	7.48%
C6	0.01%	0.07%	0.03%	0.02%	0.11%	0.00%	0.00%	0.00%

Thermoleophilia	0.01%	0.02%	0.03%	0.07%	0.03%	0.01%	0.00%	0.02%
0319-6E2	0.00%	0.00%	0.00%	0.02%	0.15%	0.02%	0.00%	0.00%
NPL-UPA2	0.00%	0.00%	0.00%	0.01%	0.11%	0.01%	0.00%	0.00%
OPB56	0.00%	0.09%	0.03%	0.02%	0.11%	0.00%	0.00%	0.00%
SJA-4	0.02%	0.03%	0.01%	0.02%	0.01%	0.02%	0.02%	0.04%
Spirochaetes	0.02%	0.03%	0.00%	0.32%	0.25%	0.00%	0.01%	0.02%
Verruco-5	0.00%	0.00%	0.00%	0.14%	0.06%	0.00%	0.00%	0.00%
Leptospirae	0.02%	0.03%	0.00%	0.05%	0.09%	0.00%	0.01%	0.02%
Erysipelotrichi	0.00%	0.00%	0.14%	0.00%	0.00%	0.00%	0.10%	0.00%
Verrucomicrobiae	0.05%	0.03%	0.05%	0.00%	0.00%	0.00%	0.05%	0.01%
Coriobacteriia	0.08%	0.01%	0.04%	0.00%	0.01%	0.00%	0.01%	0.00%
BD7-11	0.00%	0.00%	0.00%	0.00%	0.06%	0.00%	0.01%	0.00%
Fimbriimonadia	0.00%	0.01%	0.01%	0.00%	0.00%	0.04%	0.00%	0.00%
Acidobacteriia	0.01%	0.01%	0.01%	0.00%	0.00%	0.01%	0.06%	0.01%
Chlamydiia	0.02%	0.01%	0.01%	0.00%	0.00%	0.00%	0.05%	0.02%
Chloroplast	0.02%	0.00%	0.00%	0.00%	0.05%	0.00%	0.03%	0.00%
SC72	0.04%	0.00%	0.02%	0.00%	0.00%	0.00%	0.01%	0.00%
VC2_1_Bac22	0.00%	0.01%	0.00%	0.01%	0.06%	0.00%	0.00%	0.00%
Pedosphaerae	0.00%	0.00%	0.00%	0.02%	0.05%	0.00%	0.00%	0.00%
Spartobacteria	0.00%	0.00%	0.00%	0.00%	0.07%	0.00%	0.00%	0.00%
OPB41	0.05%	0.00%	0.00%	0.00%	0.00%	0.00%	0.00%	0.00%
TSBW08	0.00%	0.00%	0.00%	0.00%	0.01%	0.00%	0.00%	0.02%
GN15	0.01%	0.03%	0.00%	0.00%	0.00%	0.00%	0.00%	0.00%
Elusimicrobia	0.02%	0.00%	0.00%	0.00%	0.00%	0.00%	0.00%	0.01%
JS1	0.00%	0.00%	0.00%	0.00%	0.02%	0.00%	0.00%	0.00%

ML635J-21	0.01%	0.01%	0.01%	0.00%	0.00%	0.00%	0.00%	0.00%
DA052	0.00%	0.00%	0.00%	0.01%	0.00%	0.00%	0.01%	0.01%
SM2F11	0.00%	0.03%	0.00%	0.00%	0.00%	0.00%	0.00%	0.00%
Lentisphaeria	0.00%	0.00%	0.00%	0.00%	0.02%	0.00%	0.00%	0.00%
BSV26	0.01%	0.01%	0.00%	0.00%	0.00%	0.00%	0.00%	0.00%
Methylacidiphilae	0.00%	0.00%	0.00%	0.00%	0.02%	0.00%	0.00%	0.00%
ZB2	0.00%	0.00%	0.00%	0.00%	0.00%	0.00%	0.00%	0.00%
SHA-109	0.02%	0.00%	0.00%	0.00%	0.00%	0.00%	0.00%	0.00%
SBRH58	0.01%	0.01%	0.00%	0.00%	0.00%	0.00%	0.00%	0.00%
Holophagae	0.00%	0.00%	0.00%	0.00%	0.01%	0.00%	0.00%	0.00%
OP8_1	0.00%	0.00%	0.00%	0.00%	0.00%	0.00%	0.00%	0.00%
MVP-15	0.00%	0.00%	0.00%	0.01%	0.00%	0.00%	0.00%	0.00%
BD1-5	0.00%	0.01%	0.00%	0.00%	0.00%	0.00%	0.00%	0.00%
Gemm-5	0.00%	0.00%	0.00%	0.00%	0.00%	0.00%	0.00%	0.00%
Fusobacteriia	0.00%	0.01%	0.00%	0.00%	0.00%	0.00%	0.00%	0.00%
MB-A2-108	0.00%	0.00%	0.01%	0.00%	0.00%	0.00%	0.00%	0.00%
OPB46	0.00%	0.00%	0.00%	0.00%	0.00%	0.00%	0.00%	0.00%
B142	0.00%	0.01%	0.00%	0.00%	0.00%	0.00%	0.01%	0.00%

Table S7: Activated sludge bacterial community composition at the order level (%)

TaxonName	QG(Ano)	QG(Ana)	QG(O)	SX-1-(A)	SX-1-(O)	SX.2.OD	SY(A)	SY(O)
Nitrospirales	2.93%	3.59%	3.69%	0.34%	1.34%	58.20%	2.25%	4.49%
RB41	3.57%	10.43%	7.30%	0.82%	2.55%	1.20%	34.68%	44.19%

Burkholderiales	6.21%	9.39%	5.69%	17.76%	15.89%	7.10%	4.14%	6.98%
Sphingobacteriales	1.60%	1.98%	0.82%	0.72%	13.84%	7.31%	0.03%	0.04%
AKYG1722	10.35%	0.33%	2.04%	10.14%	4.17%	2.74%	3.09%	4.74%
Rhizobiales	6.73%	4.00%	4.35%	2.93%	5.17%	4.66%	2.01%	3.51%
Hydrogenophilales	0.07%	0.03%	0.01%	12.72%	2.18%	0.38%	1.88%	4.41%
Xanthomonadales	1.42%	2.46%	1.31%	11.64%	9.23%	0.65%	0.56%	0.92%
Caldilineales	11.20%	6.99%	14.98%	0.13%	0.47%	0.61%	0.01%	0.06%
Saprospirales	2.37%	6.74%	3.84%	0.31%	7.40%	1.32%	0.39%	0.63%
Clostridiales	1.64%	0.93%	5.78%	1.60%	0.85%	0.38%	21.90%	0.20%
SHA-20	8.50%	12.88%	9.66%	0.01%	0.00%	0.01%	0.00%	0.00%
Rhodocyclales	2.64%	2.33%	1.09%	7.09%	2.77%	1.71%	0.25%	0.72%
iii1-15	1.45%	3.74%	2.98%	0.20%	2.78%	1.38%	1.92%	1.81%
Deinococcales	0.03%	0.13%	0.02%	0.03%	0.04%	0.23%	7.70%	11.54%
Rhodospirillales	3.41%	1.33%	2.19%	1.83%	2.49%	0.99%	1.11%	1.68%
Rhodobacterales	2.64%	2.63%	2.14%	1.12%	2.89%	0.94%	1.03%	1.40%
mle1-48	2.50%	3.74%	3.74%	0.10%	0.87%	0.93%	0.89%	0.92%
Acidimicrobiales	4.30%	3.20%	2.94%	0.98%	1.66%	0.61%	0.10%	0.22%
Bacteroidales	0.61%	0.44%	2.98%	1.82%	0.92%	0.34%	6.28%	0.10%
ASSO-13	0.06%	0.03%	0.04%	2.73%	1.34%	2.39%	0.16%	0.13%
Synergistales	0.53%	0.38%	1.18%	6.75%	1.16%	0.05%	0.04%	0.02%
Actinomycetales	2.99%	1.71%	1.82%	0.31%	0.44%	0.34%	1.68%	2.34%
Ellin6067	0.99%	1.07%	0.54%	1.17%	2.64%	0.56%	0.50%	1.12%
GN03	0.04%	0.03%	0.01%	6.03%	1.82%	0.01%	0.22%	0.11%
Sphingomonadales	2.06%	0.83%	1.05%	0.91%	1.78%	0.44%	0.63%	1.22%
SBR1031	1.61%	1.02%	1.24%	0.01%	0.10%	0.62%	1.41%	1.75%

JG30-KF-CM45	1.90%	0.45%	0.99%	1.88%	0.55%	0.05%	0.77%	0.84%
SC-I-84	1.39%	2.19%	1.33%	0.19%	0.39%	0.36%	0.00%	0.01%
HOC36	2.05%	1.59%	1.30%	0.21%	0.40%	0.05%	0.00%	0.00%
SJA-15	2.22%	1.22%	1.52%	0.00%	0.00%	0.00%	0.00%	0.00%
Flavobacteriales	0.34%	0.98%	0.76%	0.08%	0.38%	0.16%	0.19%	0.31%
Legionellales	0.36%	0.17%	0.12%	0.15%	0.75%	0.55%	0.07%	0.14%
Nitrosomonadales	0.03%	0.03%	0.01%	0.36%	1.31%	0.27%	0.08%	0.17%
Chromatiales	0.63%	0.41%	0.19%	0.08%	0.32%	0.13%	0.24%	0.50%
CCU21	0.72%	1.62%	1.17%	0.00%	0.00%	0.00%	0.00%	0.00%
Lactobacillales	0.44%	0.10%	0.98%	0.28%	0.32%	0.03%	0.55%	0.07%
Myxococcales	0.41%	1.00%	0.29%	0.01%	0.34%	0.07%	0.07%	0.12%
SJA-36	0.09%	0.20%	0.13%	0.82%	0.87%	0.00%	0.00%	0.00%
Ignavibacteriales	0.20%	0.17%	0.09%	0.39%	0.45%	0.22%	0.14%	0.15%
Solibacterales	0.09%	0.23%	0.06%	0.29%	0.36%	0.14%	0.17%	0.38%
S0208	0.04%	0.21%	0.04%	0.01%	0.01%	0.16%	0.00%	0.00%
Alteromonadales	0.45%	0.75%	0.42%	0.10%	0.22%	0.03%	0.08%	0.17%
Anaerolineales	1.65%	0.20%	0.37%	0.00%	0.02%	0.00%	0.00%	0.00%
MLE1-12	0.01%	0.02%	0.01%	0.11%	0.59%	0.09%	0.23%	0.14%
Desulfobacterales	0.06%	0.05%	0.19%	1.00%	0.37%	0.01%	0.00%	0.01%
Sediment-1	0.13%	0.41%	0.11%	0.00%	0.00%	0.01%	0.00%	0.00%
Caulobacterales	0.28%	0.08%	0.09%	0.15%	0.34%	0.06%	0.23%	0.40%
Syntrophobacterales	0.17%	0.11%	0.09%	0.08%	0.06%	0.18%	0.29%	0.41%
Kiloniellales	0.05%	0.07%	0.02%	0.12%	0.35%	0.15%	0.09%	0.07%
Pseudomonadales	0.25%	0.03%	0.24%	0.43%	0.23%	0.03%	0.04%	0.05%
CL500-15	0.08%	0.05%	0.04%	0.10%	0.64%	0.00%	0.00%	0.02%

Sva0725	1.06%	0.42%	0.73%	0.00%	0.00%	0.08%	0.00%	0.00%
DS-100	0.02%	1.20%	0.67%	0.00%	0.00%	0.00%	0.00%	0.00%
Desulfuromonadales	0.00%	0.00%	0.00%	0.59%	0.45%	0.00%	0.00%	0.00%
Desulfovibrionales	0.01%	0.03%	0.11%	0.59%	0.08%	0.00%	0.23%	0.01%
A31	0.05%	0.47%	0.86%	0.00%	0.00%	0.00%	0.00%	0.00%
B97	0.04%	0.13%	0.16%	0.11%	0.40%	0.00%	0.09%	0.01%
AKYG885	0.06%	0.02%	0.08%	0.01%	0.18%	0.16%	0.05%	0.06%
Ellin329	0.04%	0.03%	0.01%	0.04%	0.10%	0.06%	0.07%	0.09%
H39	0.19%	0.48%	0.37%	0.00%	0.00%	0.02%	0.00%	0.00%
Gemmatales	0.12%	0.15%	0.42%	0.07%	0.11%	0.00%	0.00%	0.00%
Campylobacterales	0.00%	0.00%	0.07%	0.28%	0.09%	0.01%	0.35%	0.00%
Bdellovibrionales	0.07%	0.06%	0.03%	0.02%	0.24%	0.02%	0.05%	0.09%
Methylococcales	0.14%	0.11%	0.24%	0.00%	0.00%	0.13%	0.00%	0.00%
CV90	0.02%	0.05%	0.11%	0.00%	0.04%	0.01%	0.04%	0.02%
Cytophagales	0.03%	0.13%	0.02%	0.01%	0.11%	0.12%	0.01%	0.01%
Rickettsiales	0.09%	0.10%	0.04%	0.01%	0.05%	0.15%	0.02%	0.01%
Oceanospirillales	0.06%	0.02%	0.01%	0.04%	0.28%	0.01%	0.05%	0.08%
BD7-3	0.00%	0.00%	0.00%	0.03%	0.29%	0.02%	0.04%	0.05%
Phycisphaerales	0.01%	0.02%	0.00%	0.13%	0.04%	0.06%	0.09%	0.03%
Thiotrichales	0.05%	0.08%	0.16%	0.01%	0.07%	0.01%	0.06%	0.01%
GCA004	0.37%	0.08%	0.17%	0.00%	0.00%	0.00%	0.00%	0.00%
PYR10d3	0.01%	0.00%	0.00%	0.09%	0.16%	0.02%	0.02%	0.01%
Roseiflexales	0.03%	0.24%	0.09%	0.00%	0.04%	0.03%	0.00%	0.00%
PHOS-HD29	0.02%	0.01%	0.03%	0.00%	0.02%	0.03%	0.07%	0.04%
d113	0.01%	0.11%	0.05%	0.03%	0.14%	0.00%	0.00%	0.00%

pLW-97	0.06%	0.33%	0.16%	0.00%	0.00%	0.00%	0.00%	0.00%
Enterobacteriales	0.05%	0.05%	0.19%	0.03%	0.01%	0.03%	0.00%	0.00%
Solirubrobacterales	0.02%	0.01%	0.03%	0.08%	0.04%	0.03%	0.00%	0.03%
Bifidobacteriales	0.07%	0.06%	0.17%	0.02%	0.01%	0.00%	0.05%	0.02%
Spirochaetales	0.00%	0.00%	0.00%	0.16%	0.10%	0.00%	0.00%	0.00%
Leptospirales	0.03%	0.04%	0.01%	0.06%	0.11%	0.00%	0.01%	0.02%
Erysipelotrichales	0.00%	0.00%	0.22%	0.00%	0.00%	0.00%	0.13%	0.00%
PK29	0.03%	0.12%	0.11%	0.00%	0.03%	0.00%	0.00%	0.00%
WCHB1-50	0.03%	0.19%	0.13%	0.00%	0.00%	0.00%	0.00%	0.00%
Verrucomicrobiales	0.08%	0.05%	0.07%	0.00%	0.00%	0.00%	0.07%	0.01%
KD8-87	0.00%	0.02%	0.00%	0.00%	0.10%	0.00%	0.05%	0.01%
EW055	0.06%	0.05%	0.09%	0.01%	0.00%	0.01%	0.00%	0.00%
envOPS12	0.13%	0.05%	0.06%	0.00%	0.00%	0.00%	0.00%	0.00%
Coriobacteriales	0.12%	0.02%	0.06%	0.00%	0.01%	0.00%	0.01%	0.00%
WCHB1-41	0.00%	0.00%	0.00%	0.15%	0.01%	0.00%	0.00%	0.00%
NB1-j	0.00%	0.03%	0.00%	0.01%	0.04%	0.01%	0.01%	0.04%
Fimbriimonadales	0.00%	0.02%	0.01%	0.00%	0.00%	0.06%	0.00%	0.00%
Acidobacteriales	0.02%	0.01%	0.01%	0.00%	0.00%	0.01%	0.08%	0.02%
Gallionellales	0.00%	0.00%	0.00%	0.09%	0.03%	0.00%	0.00%	0.00%
Bacillales	0.01%	0.00%	0.00%	0.01%	0.03%	0.03%	0.01%	0.00%
Chlamydiales	0.03%	0.01%	0.02%	0.00%	0.00%	0.00%	0.06%	0.03%
I025	0.03%	0.03%	0.05%	0.00%	0.02%	0.00%	0.00%	0.00%
Thermales	0.01%	0.03%	0.02%	0.01%	0.04%	0.00%	0.01%	0.00%
Pedosphaerales	0.00%	0.00%	0.00%	0.02%	0.06%	0.00%	0.00%	0.00%
LD1-PB3	0.00%	0.00%	0.00%	0.02%	0.07%	0.00%	0.00%	0.00%

Chthoniobacterales	0.00%	0.00%	0.00%	0.00%	0.09%	0.00%	0.00%	0.00%
Thiobacterales	0.04%	0.03%	0.01%	0.00%	0.00%	0.00%	0.00%	0.00%
Streptophyta	0.00%	0.00%	0.00%	0.00%	0.06%	0.00%	0.04%	0.00%
agg27	0.00%	0.01%	0.02%	0.00%	0.00%	0.00%	0.06%	0.01%
WCHB1-15	0.05%	0.00%	0.00%	0.00%	0.00%	0.00%	0.01%	0.00%
Blgi18	0.06%	0.00%	0.01%	0.00%	0.00%	0.00%	0.00%	0.00%
Aeromonadales	0.01%	0.01%	0.02%	0.01%	0.00%	0.00%	0.02%	0.00%
HTCC2188	0.00%	0.00%	0.00%	0.03%	0.02%	0.00%	0.00%	0.00%
YJF2-48	0.00%	0.01%	0.00%	0.00%	0.00%	0.00%	0.00%	0.04%
Gaiellales	0.00%	0.02%	0.01%	0.00%	0.00%	0.00%	0.00%	0.00%
34P16	0.00%	0.00%	0.00%	0.02%	0.02%	0.00%	0.00%	0.00%
Spirobacillales	0.00%	0.00%	0.00%	0.00%	0.01%	0.02%	0.00%	0.00%
FAC87	0.00%	0.01%	0.01%	0.00%	0.02%	0.00%	0.00%	0.00%
BA021	0.00%	0.00%	0.00%	0.00%	0.03%	0.00%	0.00%	0.00%
GMD14H09	0.00%	0.00%	0.00%	0.00%	0.03%	0.00%	0.00%	0.00%
CW040	0.00%	0.00%	0.01%	0.00%	0.00%	0.00%	0.04%	0.00%
Ellin6513	0.00%	0.00%	0.00%	0.01%	0.00%	0.00%	0.02%	0.01%
FAC88	0.00%	0.00%	0.00%	0.00%	0.00%	0.00%	0.00%	0.01%
Sphaerobacterales	0.00%	0.00%	0.00%	0.01%	0.01%	0.00%	0.00%	0.00%
S-BQ2-57	0.00%	0.00%	0.00%	0.00%	0.03%	0.00%	0.00%	0.00%
CFB-26	0.00%	0.01%	0.01%	0.01%	0.01%	0.00%	0.00%	0.00%
PK329	0.02%	0.02%	0.00%	0.00%	0.00%	0.00%	0.00%	0.00%
Neisseriales	0.01%	0.00%	0.00%	0.00%	0.02%	0.00%	0.00%	0.00%
Z20	0.00%	0.00%	0.00%	0.00%	0.03%	0.00%	0.00%	0.00%
Chloroflexales	0.01%	0.01%	0.01%	0.00%	0.00%	0.00%	0.00%	0.00%

Stramenopiles	0.03%	0.00%	0.00%	0.00%	0.00%	0.00%	0.00%	0.00%
YS2	0.01%	0.00%	0.00%	0.00%	0.00%	0.00%	0.02%	0.00%
Holophagales	0.00%	0.00%	0.00%	0.00%	0.01%	0.00%	0.00%	0.01%
Turicibacterales	0.00%	0.00%	0.00%	0.00%	0.01%	0.00%	0.00%	0.00%
S1198	0.00%	0.00%	0.00%	0.01%	0.00%	0.00%	0.00%	0.00%
OPB95	0.00%	0.00%	0.01%	0.00%	0.01%	0.00%	0.00%	0.00%
PL-11B10	0.00%	0.00%	0.00%	0.01%	0.01%	0.00%	0.00%	0.00%
A-2AF	0.01%	0.00%	0.01%	0.00%	0.00%	0.00%	0.00%	0.00%
Methylophilales	0.00%	0.00%	0.00%	0.00%	0.01%	0.00%	0.00%	0.00%
Ellin5290	0.00%	0.00%	0.00%	0.00%	0.00%	0.00%	0.00%	0.01%
Gemmatimonadales	0.00%	0.02%	0.00%	0.00%	0.00%	0.00%	0.00%	0.00%
MVP-88	0.01%	0.00%	0.00%	0.00%	0.00%	0.00%	0.00%	0.00%
RF32	0.00%	0.00%	0.00%	0.00%	0.00%	0.00%	0.02%	0.00%
MND1	0.00%	0.01%	0.00%	0.00%	0.00%	0.00%	0.00%	0.00%
MIZ46	0.01%	0.00%	0.00%	0.00%	0.00%	0.00%	0.00%	0.00%
Fusobacteriales	0.00%	0.01%	0.00%	0.00%	0.00%	0.00%	0.00%	0.00%
SHA-1	0.00%	0.00%	0.00%	0.00%	0.00%	0.00%	0.00%	0.00%
DS-18	0.00%	0.00%	0.00%	0.00%	0.00%	0.00%	0.00%	0.01%

Table S8: Activated sludge bacterial community composition at the family level (%)

TaxonName	QG(Ano)	QG(Ana)	QG(O)	SX-1-(A)	SX-1-(O)	SX.2.OD	SY(A)	SY(O)
Nitrospiraceae	4.92%	5.66%	5.44%	0.43%	1.99%	70.58%	2.85%	5.02%
Ellin6075	5.98%	16.44%	10.77%	1.05%	3.79%	1.45%	43.99%	49.47%
Comamonadaceae	10.03%	13.46%	7.71%	21.85%	23.18%	8.21%	4.92%	7.35%

Hydrogenophilaceae	0.12%	0.05%	0.01%	16.20%	3.24%	0.46%	2.39%	4.94%
Caldilineaceae	18.78%	11.01%	22.10%	0.16%	0.69%	0.74%	0.01%	0.07%
Rhodocyclaceae	4.42%	3.67%	1.61%	9.03%	4.11%	2.07%	0.32%	0.81%
Saprospiraceae	2.85%	8.80%	3.95%	0.30%	10.21%	1.12%	0.41%	0.56%
Trueperaceae	0.04%	0.20%	0.03%	0.04%	0.05%	0.28%	9.76%	12.92%
Hyphomicrobiaceae	3.93%	1.66%	1.94%	1.18%	3.35%	3.76%	1.15%	1.92%
Xanthomonadaceae	0.74%	1.96%	0.96%	8.45%	9.65%	0.12%	0.11%	0.13%
Sinobacteraceae	1.64%	1.92%	0.96%	6.37%	4.06%	0.68%	0.61%	0.90%
mb2424	2.05%	4.79%	3.75%	0.11%	0.91%	0.84%	1.22%	0.75%
KSB4	0.05%	0.05%	0.01%	7.68%	2.70%	0.02%	0.02%	0.03%
Rhodobacteraceae	3.63%	2.86%	2.68%	1.29%	3.25%	0.34%	0.18%	0.25%
Rhodospirillaceae	4.62%	1.52%	2.50%	0.63%	0.93%	0.73%	0.30%	0.64%
A4b	1.53%	1.44%	1.50%	0.01%	0.07%	0.74%	1.65%	1.91%
Sphingomonadaceae	2.95%	1.14%	1.38%	1.01%	2.24%	0.46%	0.70%	1.17%
RB40	0.12%	0.45%	0.22%	0.10%	2.77%	0.57%	1.09%	1.13%
Hyphomonadaceae	0.79%	1.29%	0.49%	0.15%	1.05%	0.80%	1.13%	1.32%
Acetobacteraceae	0.73%	0.35%	0.45%	1.57%	2.29%	0.36%	1.08%	1.21%
Ruminococcaceae	0.24%	0.22%	2.16%	0.12%	0.14%	0.16%	10.27%	0.02%
C111	3.86%	1.84%	1.82%	0.44%	1.32%	0.34%	0.01%	0.09%
Dethiosulfovibrionaceae	0.23%	0.18%	0.41%	4.60%	0.79%	0.02%	0.02%	0.01%
Synergistaceae	0.47%	0.32%	1.16%	3.23%	0.84%	0.02%	0.02%	0.01%
Microbacteriaceae	0.55%	0.20%	0.22%	0.16%	0.26%	0.26%	1.56%	2.19%
Bradyrhizobiaceae	1.42%	0.72%	0.52%	0.86%	0.99%	0.09%	0.68%	0.82%
Methylocystaceae	2.62%	1.82%	1.89%	0.36%	0.59%	0.13%	0.01%	0.09%
S24-7	0.07%	0.03%	2.72%	0.00%	0.23%	0.00%	4.44%	0.01%

Chitinophagaceae	1.11%	1.77%	1.66%	0.01%	0.53%	0.45%	0.08%	0.14%
Nitrosomonadaceae	0.04%	0.05%	0.02%	0.46%	1.94%	0.33%	0.10%	0.19%
Cryomorphaceae	0.47%	1.54%	0.65%	0.09%	0.44%	0.18%	0.21%	0.32%
Ignavibacteriaceae	0.34%	0.27%	0.14%	0.49%	0.67%	0.27%	0.18%	0.17%
Anaerolinaceae	2.77%	0.32%	0.54%	0.00%	0.02%	0.00%	0.00%	0.00%
Intrasporangiaceae	1.35%	1.19%	1.24%	0.01%	0.02%	0.00%	0.00%	0.00%
Veillonellaceae	0.62%	0.57%	1.33%	0.11%	0.30%	0.06%	0.19%	0.03%
Caulobacteraceae	0.47%	0.13%	0.13%	0.20%	0.51%	0.07%	0.29%	0.45%
Lachnospiraceae	0.12%	0.08%	0.50%	0.05%	0.14%	0.18%	1.50%	0.09%
125ds10	0.70%	1.19%	0.60%	0.05%	0.02%	0.01%	0.07%	0.19%
Syntrophobacteraceae	0.15%	0.17%	0.13%	0.08%	0.07%	0.22%	0.36%	0.46%
Streptococcaceae	0.52%	0.10%	0.51%	0.29%	0.32%	0.03%	0.32%	0.06%
Desulfobulbaceae	0.09%	0.08%	0.29%	0.95%	0.34%	0.00%	0.00%	0.01%
Clostridiaceae	0.26%	0.20%	0.76%	0.43%	0.15%	0.02%	0.11%	0.02%
EB1017	1.02%	1.07%	0.62%	0.00%	0.00%	0.01%	0.00%	0.00%
CV106	0.00%	0.00%	0.00%	0.00%	0.00%	0.00%	0.00%	0.00%
Geobacteraceae	0.00%	0.00%	0.00%	0.70%	0.67%	0.00%	0.00%	0.00%
Solibacteraceae	0.00%	0.03%	0.00%	0.34%	0.48%	0.04%	0.08%	0.21%
Bacteroidaceae	0.08%	0.05%	0.30%	0.02%	0.05%	0.36%	0.29%	0.05%
Mogibacteriaceae	0.58%	0.08%	0.36%	0.43%	0.14%	0.02%	0.00%	0.01%
Phyllobacteriaceae	0.23%	0.18%	0.15%	0.11%	0.31%	0.08%	0.07%	0.17%
S47	0.08%	0.74%	1.27%	0.00%	0.00%	0.00%	0.00%	0.00%
Erythrobacteraceae	0.18%	0.02%	0.01%	0.14%	0.41%	0.07%	0.10%	0.19%
Tissierellaceae	0.06%	0.05%	0.07%	0.73%	0.20%	0.00%	0.00%	0.03%
Chromatiaceae	0.91%	0.47%	0.25%	0.06%	0.08%	0.02%	0.00%	0.00%

Rhizobiaceae	0.14%	0.05%	0.14%	0.09%	0.47%	0.05%	0.04%	0.09%
Coxiellaceae	0.19%	0.03%	0.04%	0.02%	0.13%	0.32%	0.02%	0.06%
SHA-31	1.05%	0.10%	0.14%	0.01%	0.08%	0.02%	0.10%	0.05%
Pseudomonadaceae	0.35%	0.03%	0.05%	0.41%	0.16%	0.03%	0.05%	0.05%
Dolo_23	0.11%	0.03%	0.12%	0.01%	0.27%	0.20%	0.06%	0.07%
Prevotellaceae	0.03%	0.10%	0.30%	0.22%	0.32%	0.03%	0.17%	0.00%
Microthrixaceae	0.72%	0.54%	0.29%	0.02%	0.06%	0.00%	0.00%	0.01%
SB-1	0.05%	0.03%	0.00%	0.63%	0.21%	0.00%	0.00%	0.02%
Haliangiaceae	0.33%	0.40%	0.17%	0.01%	0.12%	0.02%	0.00%	0.00%
Rikenellaceae	0.00%	0.02%	0.14%	0.11%	0.02%	0.00%	1.08%	0.00%
Xanthobacteraceae	0.08%	0.00%	0.04%	0.31%	0.32%	0.03%	0.00%	0.01%
Lactobacillaceae	0.03%	0.00%	0.68%	0.01%	0.14%	0.00%	0.39%	0.01%
Bdellovibrionaceae	0.12%	0.10%	0.04%	0.03%	0.35%	0.02%	0.05%	0.10%
Desulfomicrobiaceae	0.01%	0.02%	0.00%	0.68%	0.07%	0.00%	0.00%	0.01%
Mycobacteriaceae	0.16%	0.10%	0.02%	0.04%	0.04%	0.01%	0.27%	0.23%
Beijerinckiaceae	0.29%	0.05%	0.06%	0.01%	0.04%	0.23%	0.00%	0.00%
Cytophagaceae	0.04%	0.20%	0.03%	0.02%	0.14%	0.14%	0.01%	0.01%
Helicobacteraceae	0.00%	0.00%	0.07%	0.34%	0.07%	0.00%	0.45%	0.00%
Burkholderiaceae	0.17%	0.32%	0.18%	0.01%	0.02%	0.15%	0.00%	0.00%
Halomonadaceae	0.09%	0.03%	0.01%	0.06%	0.42%	0.02%	0.06%	0.09%
Legionellaceae	0.23%	0.03%	0.02%	0.01%	0.04%	0.11%	0.06%	0.09%
Peptostreptococcaceae	0.11%	0.03%	0.78%	0.04%	0.02%	0.01%	0.01%	0.01%
Nocardioidaceae	0.48%	0.17%	0.28%	0.01%	0.04%	0.01%	0.02%	0.01%
PRR-10	0.19%	0.64%	0.15%	0.00%	0.00%	0.00%	0.00%	0.00%
Moraxellaceae	0.07%	0.02%	0.31%	0.14%	0.19%	0.01%	0.00%	0.01%

Marinilabiaceae	0.02%	0.00%	0.00%	0.47%	0.11%	0.00%	0.00%	0.00%
Isosphaeraceae	0.17%	0.17%	0.51%	0.04%	0.04%	0.00%	0.00%	0.00%
Porphyromonadaceae	0.08%	0.02%	0.41%	0.11%	0.05%	0.02%	0.08%	0.00%
Desulfobacteraceae	0.00%	0.00%	0.00%	0.33%	0.21%	0.00%	0.00%	0.00%
Methylococcaceae	0.12%	0.10%	0.12%	0.00%	0.00%	0.16%	0.00%	0.00%
GZKB119	0.00%	0.00%	0.00%	0.37%	0.16%	0.00%	0.00%	0.00%
Propionibacteriaceae	0.34%	0.03%	0.15%	0.01%	0.15%	0.00%	0.01%	0.01%
Piscirickettsiaceae	0.07%	0.10%	0.03%	0.01%	0.11%	0.01%	0.07%	0.01%
Weeksellaceae	0.02%	0.00%	0.45%	0.01%	0.05%	0.00%	0.01%	0.01%
TTA_B6	0.16%	0.07%	0.14%	0.19%	0.05%	0.00%	0.00%	0.01%
Alteromonadaceae	0.03%	0.00%	0.01%	0.08%	0.25%	0.02%	0.02%	0.00%
Desulfovibrionaceae	0.00%	0.03%	0.17%	0.07%	0.05%	0.00%	0.29%	0.01%
Alcaligenaceae	0.00%	0.05%	0.05%	0.01%	0.13%	0.08%	0.02%	0.01%
Enterobacteriaceae	0.08%	0.08%	0.29%	0.04%	0.01%	0.04%	0.00%	0.00%
Bifidobacteriaceae	0.12%	0.10%	0.25%	0.03%	0.02%	0.00%	0.06%	0.02%
PAUC26f	0.00%	0.00%	0.00%	0.01%	0.00%	0.04%	0.08%	0.11%
Aminiphilaceae	0.01%	0.00%	0.00%	0.29%	0.04%	0.00%	0.00%	0.00%
Spirochaetaceae	0.00%	0.00%	0.00%	0.20%	0.14%	0.00%	0.00%	0.00%
Thermovirgaceae	0.00%	0.00%	0.00%	0.26%	0.00%	0.01%	0.00%	0.00%
Carnobacteriaceae	0.17%	0.03%	0.20%	0.04%	0.01%	0.00%	0.00%	0.00%
Erysipelotrichaceae	0.00%	0.00%	0.33%	0.00%	0.00%	0.00%	0.17%	0.00%
Gemmataceae	0.03%	0.07%	0.12%	0.05%	0.12%	0.00%	0.00%	0.00%
Oxalobacteraceae	0.00%	0.28%	0.13%	0.02%	0.02%	0.02%	0.00%	0.00%
Verrucomicrobiaceae	0.14%	0.08%	0.11%	0.00%	0.00%	0.00%	0.08%	0.01%
Crenotrichaceae	0.11%	0.07%	0.24%	0.00%	0.00%	0.00%	0.00%	0.00%

Peptococcaceae	0.07%	0.05%	0.11%	0.06%	0.02%	0.00%	0.05%	0.00%
Dietziaceae	0.25%	0.03%	0.05%	0.00%	0.02%	0.00%	0.00%	0.00%
Coriobacteriaceae	0.20%	0.03%	0.10%	0.00%	0.02%	0.00%	0.01%	0.00%
Methylobacteriaceae	0.02%	0.00%	0.00%	0.10%	0.02%	0.03%	0.04%	0.00%
RFP12	0.00%	0.00%	0.00%	0.19%	0.02%	0.00%	0.00%	0.00%
Nakamurellaceae	0.00%	0.00%	0.00%	0.00%	0.00%	0.00%	0.13%	0.10%
Flavobacteriaceae	0.07%	0.00%	0.02%	0.01%	0.05%	0.01%	0.02%	0.03%
JTB38	0.00%	0.05%	0.00%	0.01%	0.06%	0.02%	0.01%	0.02%
AKIW874	0.00%	0.00%	0.00%	0.00%	0.00%	0.10%	0.00%	0.00%
Christensenellaceae	0.14%	0.03%	0.06%	0.02%	0.01%	0.01%	0.00%	0.00%
Kouleothrixaceae	0.00%	0.33%	0.07%	0.00%	0.00%	0.00%	0.00%	0.00%
Roseiflexaceae	0.00%	0.00%	0.01%	0.00%	0.00%	0.04%	0.00%	0.00%
Ectothiorhodospiraceae	0.09%	0.08%	0.02%	0.00%	0.00%	0.03%	0.00%	0.00%
Gallionellaceae	0.00%	0.00%	0.00%	0.12%	0.05%	0.00%	0.00%	0.00%
Leptospiraceae	0.04%	0.07%	0.01%	0.01%	0.09%	0.00%	0.00%	0.01%
Paraprevotellaceae	0.02%	0.00%	0.01%	0.00%	0.00%	0.00%	0.27%	0.00%
BA008	0.01%	0.00%	0.00%	0.10%	0.06%	0.00%	0.00%	0.00%
Sediment-4	0.00%	0.00%	0.00%	0.07%	0.07%	0.00%	0.01%	0.02%
Koribacteraceae	0.03%	0.02%	0.02%	0.00%	0.00%	0.01%	0.10%	0.01%
Corynebacteriaceae	0.01%	0.03%	0.00%	0.00%	0.06%	0.02%	0.05%	0.01%
oc28	0.04%	0.07%	0.12%	0.00%	0.00%	0.00%	0.04%	0.00%
Thiotrichaceae	0.01%	0.03%	0.20%	0.00%	0.00%	0.00%	0.00%	0.00%
Myxococcaceae	0.20%	0.03%	0.00%	0.00%	0.00%	0.00%	0.00%	0.00%
Nocardiaceae	0.00%	0.00%	0.00%	0.03%	0.04%	0.00%	0.00%	0.02%
Dehalobacteriaceae	0.01%	0.00%	0.02%	0.00%	0.00%	0.00%	0.21%	0.00%

Thermaceae	0.02%	0.05%	0.03%	0.01%	0.05%	0.00%	0.01%	0.00%
Odoribacteraceae	0.00%	0.00%	0.05%	0.00%	0.00%	0.00%	0.16%	0.00%
Chthoniobacteraceae	0.00%	0.00%	0.00%	0.00%	0.13%	0.00%	0.00%	0.00%
auto67_4W	0.00%	0.00%	0.00%	0.03%	0.09%	0.00%	0.00%	0.00%
Campylobacteraceae	0.00%	0.00%	0.03%	0.02%	0.06%	0.01%	0.00%	0.00%
Iamiaceae	0.00%	0.00%	0.00%	0.02%	0.00%	0.00%	0.02%	0.03%
Fimbriimonadaceae	0.00%	0.03%	0.02%	0.00%	0.00%	0.03%	0.00%	0.00%
Actinomycetaceae	0.00%	0.03%	0.05%	0.02%	0.01%	0.00%	0.00%	0.01%
ntu14	0.00%	0.00%	0.00%	0.02%	0.02%	0.00%	0.01%	0.03%
Shewanellaceae	0.02%	0.00%	0.01%	0.00%	0.05%	0.00%	0.00%	0.00%
Bacillaceae	0.00%	0.00%	0.00%	0.00%	0.02%	0.03%	0.01%	0.00%
Nannocystaceae	0.02%	0.05%	0.05%	0.00%	0.00%	0.00%	0.00%	0.00%
Syntrophaceae	0.03%	0.00%	0.01%	0.03%	0.02%	0.00%	0.00%	0.00%
Parachlamydiaceae	0.02%	0.00%	0.00%	0.00%	0.00%	0.00%	0.05%	0.03%
Syntrophorhabdaceae	0.11%	0.00%	0.00%	0.00%	0.00%	0.00%	0.00%	0.00%
Aeromonadaceae	0.01%	0.02%	0.03%	0.02%	0.00%	0.00%	0.00%	0.00%
Pseudonocardiaceae	0.00%	0.00%	0.00%	0.03%	0.00%	0.00%	0.00%	0.01%
Phycisphaeraceae	0.00%	0.00%	0.00%	0.00%	0.00%	0.00%	0.05%	0.01%
Listeriaceae	0.01%	0.00%	0.00%	0.01%	0.02%	0.00%	0.00%	0.00%
F16	0.00%	0.00%	0.02%	0.00%	0.00%	0.00%	0.05%	0.00%
Pelobacteraceae	0.00%	0.00%	0.00%	0.04%	0.00%	0.00%	0.00%	0.00%
SJA-101	0.03%	0.00%	0.03%	0.00%	0.00%	0.00%	0.00%	0.00%
Bryobacteraceae	0.04%	0.02%	0.00%	0.00%	0.00%	0.00%	0.00%	0.00%
Streptomycetaceae	0.01%	0.00%	0.02%	0.00%	0.00%	0.01%	0.00%	0.00%
Neisseriaceae	0.02%	0.00%	0.00%	0.00%	0.02%	0.00%	0.00%	0.00%

Polyangiaceae	0.01%	0.05%	0.01%	0.00%	0.00%	0.00%	0.00%	0.00%
Acidaminobacteraceae	0.00%	0.00%	0.02%	0.00%	0.00%	0.00%	0.00%	0.02%
Cellulomonadaceae	0.00%	0.00%	0.01%	0.00%	0.00%	0.01%	0.02%	0.00%
Anaerobaculaceae	0.00%	0.00%	0.00%	0.02%	0.02%	0.00%	0.00%	0.00%
AK1AB1_02E	0.00%	0.03%	0.00%	0.00%	0.00%	0.00%	0.00%	0.00%
Gordoniaceae	0.02%	0.02%	0.00%	0.00%	0.00%	0.00%	0.00%	0.00%
Holophagaceae	0.00%	0.00%	0.00%	0.00%	0.02%	0.00%	0.00%	0.01%
Gaiellaceae	0.00%	0.00%	0.02%	0.00%	0.00%	0.00%	0.00%	0.00%
Staphylococcaceae	0.00%	0.00%	0.00%	0.00%	0.01%	0.01%	0.00%	0.00%
Turicibacteraceae	0.00%	0.00%	0.00%	0.01%	0.02%	0.00%	0.00%	0.00%
Caldicoprobacteraceae	0.03%	0.00%	0.00%	0.00%	0.00%	0.00%	0.00%	0.00%
Halothiobacillaceae	0.00%	0.00%	0.00%	0.01%	0.01%	0.00%	0.00%	0.00%
Syntrophomonadaceae	0.01%	0.02%	0.01%	0.00%	0.00%	0.00%	0.00%	0.00%
Leuconostocaceae	0.01%	0.00%	0.01%	0.00%	0.01%	0.00%	0.00%	0.00%
Micrococcaceae	0.00%	0.00%	0.00%	0.02%	0.00%	0.00%	0.00%	0.00%
Conexibacteraceae	0.00%	0.00%	0.00%	0.00%	0.00%	0.01%	0.00%	0.00%
Gemmatimonadaceae	0.00%	0.03%	0.00%	0.00%	0.00%	0.00%	0.00%	0.00%
Methylophilaceae	0.00%	0.00%	0.00%	0.00%	0.02%	0.00%	0.00%	0.00%
Enterococcaceae	0.01%	0.00%	0.00%	0.01%	0.00%	0.00%	0.00%	0.00%
Succinivibrionaceae	0.00%	0.00%	0.00%	0.00%	0.00%	0.00%	0.02%	0.00%
Rs-045	0.01%	0.00%	0.01%	0.00%	0.00%	0.00%	0.00%	0.00%
Acidobacteriaceae	0.00%	0.00%	0.00%	0.00%	0.00%	0.00%	0.00%	0.01%
Rickettsiaceae	0.01%	0.00%	0.01%	0.00%	0.00%	0.00%	0.00%	0.00%
Bacteriovoracaceae	0.00%	0.00%	0.00%	0.00%	0.00%	0.00%	0.01%	0.00%
JdFBGBact	0.00%	0.00%	0.00%	0.00%	0.00%	0.00%	0.00%	0.00%

Promicromonosporaceae	0.00%	0.00%	0.00%	0.01%	0.00%	0.00%	0.00%	0.00%
Eubacteriaceae	0.01%	0.00%	0.01%	0.00%	0.00%	0.00%	0.00%	0.00%
0319-6G20	0.01%	0.00%	0.00%	0.00%	0.00%	0.00%	0.00%	0.00%
Solirubrobacteraceae	0.00%	0.00%	0.00%	0.00%	0.00%	0.00%	0.00%	0.00%
Oscillochloridaceae	0.01%	0.02%	0.00%	0.00%	0.00%	0.00%	0.00%	0.00%
Chloroflexaceae	0.00%	0.00%	0.02%	0.00%	0.00%	0.00%	0.00%	0.00%
Fusobacteriaceae	0.00%	0.02%	0.00%	0.00%	0.00%	0.00%	0.00%	0.00%
Patulibacteraceae	0.00%	0.00%	0.02%	0.00%	0.00%	0.00%	0.00%	0.00%

Table S9: Activated sludge bacterial community composition at the genus level (%)

TaxonName	QG(Ano)	QG(Ana)	QG(O)	SX-1-(A)	SX-1-(O)	SX.2.OD	SY(A)	SY(O)
Nitrospira	20.92%	33.68%	23.09%	1.72%	9.93%	94.68%	10.58%	17.68%
Thiobacillus	0.25%	0.15%	0.02%	40.44%	11.72%	0.31%	8.79%	17.26%
Caldilinea	32.19%	26.16%	39.15%	0.05%	0.05%	0.37%	0.00%	0.00%
B-42	0.09%	0.60%	0.07%	0.09%	0.19%	0.19%	35.94%	45.12%
Hyphomicrobium	5.11%	3.04%	2.56%	1.36%	7.97%	1.70%	3.39%	4.22%
Thauera	0.90%	1.40%	0.27%	7.48%	9.55%	0.15%	0.36%	1.27%
HA73	0.09%	0.10%	0.04%	11.42%	2.83%	0.01%	0.09%	0.02%
vadinCA02	1.01%	0.95%	2.47%	8.07%	3.02%	0.01%	0.09%	0.05%
Dokdonella	0.36%	3.54%	0.94%	1.11%	8.87%	0.01%	0.09%	0.31%
Alicycliphilus	0.09%	0.20%	0.09%	1.72%	3.29%	0.07%	1.43%	2.40%
Dechloromonas	6.59%	3.29%	1.15%	0.92%	0.27%	0.10%	0.04%	0.07%
Comamonas	1.90%	0.55%	0.49%	1.25%	1.36%	0.14%	1.16%	1.37%
Rhodobacter	2.55%	4.33%	2.68%	0.49%	1.88%	0.00%	0.00%	0.02%
Oscillospira	0.02%	0.00%	0.88%	0.03%	0.05%	0.00%	14.06%	0.02%

Rhodoplanes	0.54%	0.15%	0.13%	0.68%	1.39%	0.30%	0.09%	0.35%
Paracoccus	3.81%	0.90%	1.01%	0.40%	1.58%	0.02%	0.04%	0.09%
Novosphingobium	2.33%	0.45%	0.43%	0.13%	1.47%	0.09%	0.22%	0.26%
Hydrogenophaga	0.36%	0.80%	0.29%	1.22%	1.36%	0.05%	0.09%	0.35%
Geobacter	0.00%	0.00%	0.00%	1.74%	2.42%	0.00%	0.00%	0.00%
Bacteroides	0.18%	0.15%	0.63%	0.05%	0.16%	0.24%	1.07%	0.19%
Sphingobium	0.02%	0.20%	0.02%	0.33%	1.25%	0.05%	0.63%	0.83%
Ruminococcus	0.13%	0.10%	1.08%	0.05%	0.08%	0.01%	4.73%	0.00%
Prevotella	0.11%	0.30%	0.65%	0.55%	1.14%	0.02%	1.29%	0.00%
Pseudomonas	0.74%	0.10%	0.11%	1.01%	0.49%	0.02%	0.18%	0.19%
Steroidobacter	0.00%	0.00%	0.00%	0.49%	1.60%	0.07%	0.13%	0.21%
Tissierella_Soehngenia	0.02%	0.00%	0.02%	1.77%	0.63%	0.00%	0.00%	0.07%
Hyphomonas	0.07%	0.00%	0.00%	0.25%	2.37%	0.02%	0.22%	0.26%
Streptococcus	0.38%	0.30%	0.81%	0.51%	0.68%	0.02%	0.09%	0.00%
Desulfobulbus	0.18%	0.20%	0.61%	1.03%	0.54%	0.00%	0.00%	0.02%
Limnohabitans	0.25%	1.15%	0.36%	0.41%	0.76%	0.02%	0.00%	0.02%
Phenylobacterium	0.09%	0.10%	0.00%	0.41%	1.22%	0.00%	0.58%	0.64%
Lactobacillus	0.07%	0.00%	1.44%	0.03%	0.52%	0.00%	1.43%	0.05%
Desulfomicrobium	0.02%	0.05%	0.00%	1.71%	0.24%	0.00%	0.00%	0.02%
T78	2.26%	0.25%	0.22%	0.00%	0.08%	0.00%	0.00%	0.00%
Bdellovibrio	0.25%	0.30%	0.09%	0.08%	1.25%	0.01%	0.18%	0.33%
Sphingomonas	0.27%	0.10%	0.04%	0.02%	0.54%	0.00%	0.85%	1.32%
Anaerovorax	0.25%	0.05%	0.31%	1.06%	0.49%	0.01%	0.00%	0.02%
Mycobacterium	0.34%	0.30%	0.04%	0.11%	0.14%	0.00%	0.98%	0.80%
Xanthobacter	0.18%	0.00%	0.07%	0.76%	1.01%	0.02%	0.00%	0.00%
Lactococcus	0.72%	0.00%	0.27%	0.22%	0.46%	0.00%	1.07%	0.21%
Devosia	0.78%	0.50%	0.56%	0.05%	0.11%	0.05%	0.00%	0.09%
Mesorhizobium	0.20%	0.35%	0.20%	0.11%	0.38%	0.03%	0.18%	0.45%

Candidatus	0.18%	0.25%	0.02%	0.22%	0.57%	0.01%	0.22%	0.42%
Pedomicrobium	0.36%	0.15%	0.13%	0.00%	0.00%	0.14%	0.00%	0.00%
Leucobacter	0.45%	0.35%	0.20%	0.30%	0.60%	0.00%	0.18%	0.26%
Aquicella	0.00%	0.00%	0.02%	0.03%	0.22%	0.18%	0.00%	0.00%
Faecalibacterium	0.16%	0.20%	0.38%	0.16%	0.11%	0.09%	0.00%	0.05%
Methylibium	0.34%	0.75%	0.20%	0.05%	0.05%	0.06%	0.13%	0.09%
Allochromatium	1.21%	0.45%	0.18%	0.02%	0.08%	0.01%	0.00%	0.00%
Dok59	0.02%	1.84%	0.74%	0.06%	0.03%	0.00%	0.00%	0.00%
Thermomonas	0.34%	0.55%	0.47%	0.09%	0.11%	0.03%	0.00%	0.12%
Propionicimonas	0.92%	0.50%	0.52%	0.03%	0.00%	0.00%	0.00%	0.00%
Clostridium	0.20%	0.35%	0.56%	0.28%	0.33%	0.00%	0.00%	0.00%
Desulfococcus	0.00%	0.00%	0.00%	0.79%	0.65%	0.00%	0.00%	0.00%
Veillonella	0.18%	0.10%	0.43%	0.03%	0.79%	0.01%	0.22%	0.02%
Halomonas	0.16%	0.05%	0.00%	0.05%	1.39%	0.01%	0.13%	0.07%
Parvibaculum	0.02%	0.00%	0.00%	0.43%	0.98%	0.00%	0.04%	0.02%
E6	0.34%	0.20%	0.29%	0.47%	0.16%	0.00%	0.00%	0.02%
PD-UASB-13	0.40%	0.45%	0.79%	0.06%	0.03%	0.00%	0.00%	0.00%
Longilinea	1.10%	0.30%	0.29%	0.00%	0.00%	0.00%	0.00%	0.00%
HB2-32-21	0.07%	0.00%	0.00%	0.21%	0.87%	0.01%	0.09%	0.00%
Blastomonas	0.34%	0.05%	0.02%	0.02%	0.41%	0.03%	0.00%	0.17%
Methylocaldum	0.09%	0.00%	0.07%	0.00%	0.00%	0.10%	0.00%	0.00%
Salinibacterium	0.49%	0.15%	0.09%	0.08%	0.11%	0.01%	0.22%	0.17%
Bifidobacterium	0.25%	0.30%	0.54%	0.06%	0.05%	0.00%	0.22%	0.07%
Cloacibacterium	0.04%	0.00%	0.97%	0.00%	0.03%	0.00%	0.04%	0.02%
Phascolarctobacterium	0.22%	0.10%	0.31%	0.05%	0.05%	0.03%	0.13%	0.02%
Methylosinus	0.13%	0.40%	0.13%	0.03%	0.19%	0.01%	0.04%	0.24%
Methyloversatilis	0.00%	0.00%	0.00%	0.22%	0.14%	0.01%	0.00%	0.09%
Kaistobacter	0.00%	0.00%	0.00%	0.16%	0.49%	0.00%	0.31%	0.24%

Proteiniclasticum	0.16%	0.10%	0.63%	0.06%	0.00%	0.00%	0.09%	0.05%
Sulfuricurvum	0.00%	0.00%	0.00%	0.70%	0.05%	0.00%	0.00%	0.00%
Allobaculum	0.00%	0.00%	0.70%	0.00%	0.00%	0.00%	0.63%	0.00%
Paludibacter	0.11%	0.05%	0.63%	0.11%	0.03%	0.00%	0.00%	0.00%
Aminiphilus	0.02%	0.00%	0.00%	0.60%	0.08%	0.00%	0.00%	0.00%
Crenothrix	0.22%	0.20%	0.52%	0.00%	0.00%	0.00%	0.00%	0.00%
Dietzia	0.54%	0.10%	0.11%	0.00%	0.08%	0.00%	0.00%	0.00%
Anaerolinea	0.69%	0.00%	0.04%	0.00%	0.00%	0.00%	0.00%	0.00%
Acidovorax	0.07%	0.60%	0.25%	0.00%	0.00%	0.00%	0.00%	0.14%
Helicobacter	0.00%	0.00%	0.11%	0.00%	0.00%	0.00%	1.21%	0.00%
Burkholderia	0.00%	0.00%	0.00%	0.00%	0.03%	0.07%	0.00%	0.00%
Rubrivivax	0.31%	0.50%	0.16%	0.00%	0.00%	0.00%	0.00%	0.00%
Zoogloea	0.16%	0.50%	0.29%	0.00%	0.00%	0.00%	0.00%	0.00%
Acinetobacter	0.07%	0.05%	0.31%	0.03%	0.16%	0.00%	0.00%	0.02%
Treponema	0.00%	0.00%	0.00%	0.32%	0.24%	0.00%	0.00%	0.00%
Perlucidibaca	0.00%	0.00%	0.00%	0.22%	0.30%	0.00%	0.00%	0.00%
Megasphaera	0.02%	0.05%	0.34%	0.03%	0.14%	0.00%	0.18%	0.00%
Kouleothrix	0.00%	1.00%	0.16%	0.00%	0.00%	0.00%	0.00%	0.00%
Roseiflexus	0.00%	0.00%	0.02%	0.00%	0.00%	0.03%	0.00%	0.00%
vadinHB04	0.04%	0.25%	0.38%	0.00%	0.08%	0.00%	0.00%	0.00%
Pigmentiphaga	0.00%	0.00%	0.00%	0.00%	0.30%	0.03%	0.00%	0.00%
Kaistia	0.00%	0.00%	0.00%	0.08%	0.54%	0.00%	0.00%	0.00%
Coprococcus	0.04%	0.00%	0.29%	0.00%	0.00%	0.00%	0.36%	0.00%
Gallionella	0.00%	0.00%	0.00%	0.30%	0.16%	0.00%	0.00%	0.00%
Desulfovibrio	0.00%	0.10%	0.20%	0.09%	0.03%	0.00%	0.27%	0.02%
Bilophila	0.00%	0.00%	0.16%	0.00%	0.00%	0.00%	0.71%	0.00%
Thiothrix	0.02%	0.10%	0.43%	0.00%	0.00%	0.00%	0.00%	0.00%
Corynebacterium	0.02%	0.10%	0.00%	0.00%	0.22%	0.01%	0.18%	0.02%

Flavobacterium	0.16%	0.00%	0.00%	0.03%	0.19%	0.01%	0.00%	0.05%
Anaeromyxobacter	0.43%	0.10%	0.00%	0.00%	0.00%	0.00%	0.00%	0.00%
Lachnospira	0.00%	0.10%	0.02%	0.00%	0.00%	0.04%	0.00%	0.00%
Blvii28	0.00%	0.00%	0.00%	0.27%	0.08%	0.00%	0.00%	0.00%
Dehalobacterium	0.02%	0.00%	0.04%	0.00%	0.00%	0.00%	0.76%	0.00%
Rhodococcus	0.00%	0.00%	0.00%	0.08%	0.14%	0.00%	0.00%	0.07%
Enhydrobacter	0.07%	0.00%	0.34%	0.00%	0.03%	0.00%	0.00%	0.00%
Chryseobacterium	0.00%	0.00%	0.00%	0.00%	0.14%	0.00%	0.00%	0.00%
Turneriella	0.09%	0.05%	0.02%	0.02%	0.27%	0.00%	0.00%	0.02%
SHD-231	0.27%	0.00%	0.16%	0.00%	0.00%	0.00%	0.00%	0.00%
Odoribacter	0.00%	0.00%	0.11%	0.00%	0.00%	0.00%	0.58%	0.00%
Simplicispira	0.00%	0.25%	0.02%	0.00%	0.00%	0.00%	0.09%	0.14%
Megamonas	0.04%	0.05%	0.22%	0.00%	0.03%	0.00%	0.00%	0.00%
Arenimonas	0.00%	0.00%	0.00%	0.16%	0.19%	0.00%	0.00%	0.00%
Roseburia	0.00%	0.00%	0.00%	0.00%	0.00%	0.03%	0.00%	0.07%
Propionivibrio	0.02%	0.00%	0.07%	0.16%	0.05%	0.00%	0.00%	0.00%
Anaerospora	0.07%	0.00%	0.00%	0.03%	0.19%	0.00%	0.00%	0.00%
Tepidimonas	0.00%	0.00%	0.00%	0.09%	0.19%	0.00%	0.00%	0.02%
Parabacteroides	0.00%	0.00%	0.09%	0.00%	0.00%	0.01%	0.31%	0.00%
Diaphorobacter	0.00%	0.35%	0.13%	0.00%	0.00%	0.00%	0.00%	0.02%
Azoarcus	0.09%	0.25%	0.00%	0.06%	0.00%	0.00%	0.00%	0.00%
Iamia	0.00%	0.00%	0.00%	0.05%	0.00%	0.00%	0.09%	0.09%
Inquilinus	0.00%	0.00%	0.09%	0.00%	0.03%	0.01%	0.04%	0.00%
Sulfuritalea	0.00%	0.40%	0.02%	0.00%	0.11%	0.00%	0.04%	0.00%
Hylemonella	0.07%	0.10%	0.07%	0.03%	0.03%	0.01%	0.00%	0.00%
Tatlockia	0.00%	0.00%	0.00%	0.00%	0.00%	0.03%	0.00%	0.00%
AF12	0.00%	0.00%	0.04%	0.02%	0.00%	0.00%	0.49%	0.00%
za29	0.00%	0.00%	0.00%	0.14%	0.14%	0.00%	0.00%	0.00%

Arcobacter	0.00%	0.00%	0.07%	0.05%	0.14%	0.00%	0.00%	0.00%
Sutterella	0.00%	0.00%	0.00%	0.00%	0.00%	0.03%	0.00%	0.00%
Methylobacterium	0.04%	0.00%	0.00%	0.09%	0.00%	0.00%	0.09%	0.00%
Thermus	0.04%	0.00%	0.00%	0.03%	0.19%	0.00%	0.04%	0.00%
Legionella	0.02%	0.00%	0.00%	0.00%	0.03%	0.00%	0.09%	0.12%
Bacillus	0.00%	0.00%	0.00%	0.00%	0.05%	0.02%	0.04%	0.00%
GW-34	0.07%	0.05%	0.11%	0.00%	0.05%	0.00%	0.00%	0.00%
Pleomorphomonas	0.02%	0.00%	0.09%	0.09%	0.00%	0.00%	0.00%	0.00%
Shewanella	0.04%	0.00%	0.02%	0.00%	0.19%	0.00%	0.00%	0.00%
Nannocystis	0.04%	0.15%	0.11%	0.00%	0.00%	0.00%	0.00%	0.00%
Ramlibacter	0.02%	0.15%	0.04%	0.00%	0.03%	0.00%	0.00%	0.00%
Anaeromusa	0.00%	0.10%	0.13%	0.02%	0.00%	0.00%	0.00%	0.00%
Blautia	0.00%	0.05%	0.02%	0.05%	0.00%	0.01%	0.00%	0.00%
Haliscomenobacter	0.09%	0.05%	0.09%	0.00%	0.00%	0.00%	0.00%	0.00%
Rikenella	0.00%	0.00%	0.02%	0.00%	0.00%	0.00%	0.36%	0.00%
KD1-23	0.09%	0.15%	0.00%	0.03%	0.00%	0.00%	0.00%	0.00%
Nostocoida	0.02%	0.05%	0.13%	0.00%	0.00%	0.00%	0.00%	0.00%
Sphingopyxis	0.02%	0.05%	0.00%	0.00%	0.00%	0.01%	0.00%	0.00%
Methylopila	0.09%	0.00%	0.09%	0.00%	0.00%	0.00%	0.00%	0.00%
Fluviicola	0.00%	0.00%	0.00%	0.05%	0.05%	0.00%	0.04%	0.05%
Stenotrophomonas	0.04%	0.05%	0.02%	0.02%	0.00%	0.00%	0.00%	0.00%
Pseudonocardia	0.00%	0.00%	0.00%	0.06%	0.00%	0.00%	0.00%	0.02%
Bradyrhizobium	0.09%	0.00%	0.00%	0.03%	0.00%	0.00%	0.00%	0.00%
Brochothrix	0.02%	0.00%	0.00%	0.03%	0.08%	0.00%	0.00%	0.00%
CF231	0.00%	0.00%	0.00%	0.00%	0.00%	0.00%	0.31%	0.00%
Actinomyces	0.00%	0.05%	0.07%	0.05%	0.00%	0.00%	0.00%	0.00%
Sedimentibacter	0.00%	0.00%	0.00%	0.05%	0.05%	0.00%	0.00%	0.02%
Propionibacterium	0.00%	0.00%	0.02%	0.00%	0.08%	0.00%	0.04%	0.02%

Chelatococcus	0.00%	0.00%	0.00%	0.03%	0.14%	0.00%	0.00%	0.00%
Meiothermus	0.00%	0.15%	0.07%	0.00%	0.00%	0.00%	0.00%	0.00%
N09	0.00%	0.05%	0.04%	0.00%	0.03%	0.00%	0.00%	0.05%
Desulfomonile	0.00%	0.00%	0.00%	0.06%	0.05%	0.00%	0.00%	0.00%
Muricauda	0.00%	0.00%	0.00%	0.00%	0.00%	0.00%	0.09%	0.05%
Pseudoxanthomonas	0.02%	0.10%	0.04%	0.02%	0.00%	0.00%	0.00%	0.00%
Agrobacterium	0.07%	0.00%	0.02%	0.00%	0.03%	0.00%	0.00%	0.00%
Azospirillum	0.02%	0.00%	0.00%	0.00%	0.00%	0.00%	0.00%	0.00%
Luteibacter	0.00%	0.00%	0.02%	0.00%	0.00%	0.00%	0.22%	0.00%
Leptonema	0.00%	0.15%	0.00%	0.02%	0.05%	0.00%	0.00%	0.00%
Succiniclasticum	0.02%	0.10%	0.00%	0.05%	0.00%	0.00%	0.00%	0.00%
Roseateles	0.09%	0.05%	0.02%	0.00%	0.00%	0.00%	0.00%	0.00%
Lysobacter	0.00%	0.00%	0.07%	0.00%	0.00%	0.00%	0.00%	0.00%
Syntrophobacter	0.07%	0.00%	0.00%	0.03%	0.00%	0.00%	0.00%	0.00%
Streptomyces	0.02%	0.00%	0.04%	0.00%	0.00%	0.00%	0.00%	0.00%
C39	0.00%	0.10%	0.00%	0.03%	0.00%	0.00%	0.00%	0.00%
Actinotalea	0.00%	0.00%	0.02%	0.00%	0.00%	0.00%	0.09%	0.00%
Sulfurospirillum	0.00%	0.00%	0.00%	0.00%	0.08%	0.00%	0.00%	0.00%
Staphylococcus	0.00%	0.00%	0.00%	0.00%	0.03%	0.01%	0.00%	0.00%
Collinsella	0.02%	0.00%	0.02%	0.00%	0.05%	0.00%	0.00%	0.00%
Akkermansia	0.00%	0.00%	0.02%	0.00%	0.00%	0.00%	0.13%	0.00%
Turicibacter	0.00%	0.00%	0.00%	0.02%	0.05%	0.00%	0.00%	0.00%
Z-35	0.00%	0.00%	0.00%	0.05%	0.00%	0.00%	0.00%	0.00%
SJA-88	0.00%	0.00%	0.00%	0.00%	0.11%	0.00%	0.00%	0.00%
Gordonia	0.04%	0.05%	0.00%	0.00%	0.00%	0.00%	0.00%	0.00%
Pelotomaculum	0.00%	0.10%	0.04%	0.00%	0.00%	0.00%	0.00%	0.00%
Isosphaera	0.02%	0.05%	0.02%	0.00%	0.00%	0.00%	0.00%	0.00%
Niabella	0.02%	0.00%	0.04%	0.00%	0.00%	0.00%	0.00%	0.00%

Caldicoprobacter	0.07%	0.00%	0.00%	0.00%	0.00%	0.00%	0.00%	0.00%
Syntrophomonas	0.02%	0.05%	0.02%	0.00%	0.00%	0.00%	0.00%	0.00%
Cryocola	0.00%	0.00%	0.00%	0.00%	0.00%	0.00%	0.00%	0.05%
Gallicola	0.02%	0.05%	0.02%	0.00%	0.00%	0.00%	0.00%	0.00%
Acidaminobacter	0.00%	0.00%	0.00%	0.00%	0.00%	0.00%	0.00%	0.07%
Luteimonas	0.00%	0.00%	0.00%	0.00%	0.05%	0.00%	0.00%	0.00%
Rothia	0.00%	0.00%	0.00%	0.05%	0.00%	0.00%	0.00%	0.00%
Neisseria	0.00%	0.00%	0.00%	0.00%	0.08%	0.00%	0.00%	0.00%
Leadbetterella	0.00%	0.15%	0.00%	0.00%	0.00%	0.00%	0.00%	0.00%
Yonghaparkia	0.00%	0.00%	0.00%	0.00%	0.00%	0.00%	0.04%	0.02%
Klebsiella	0.00%	0.00%	0.04%	0.00%	0.00%	0.00%	0.00%	0.00%
Methylomonas	0.00%	0.10%	0.00%	0.00%	0.00%	0.00%	0.00%	0.00%
Prosthecobacter	0.00%	0.00%	0.00%	0.00%	0.00%	0.00%	0.04%	0.00%
Desulfobacter	0.00%	0.00%	0.00%	0.00%	0.05%	0.00%	0.00%	0.00%
Enterococcus	0.02%	0.00%	0.00%	0.02%	0.00%	0.00%	0.00%	0.00%
Clavibacter	0.00%	0.00%	0.00%	0.00%	0.00%	0.00%	0.00%	0.02%
Anaerostipes	0.00%	0.00%	0.02%	0.00%	0.00%	0.00%	0.04%	0.00%
Amaricoccus	0.04%	0.00%	0.00%	0.00%	0.00%	0.00%	0.00%	0.00%
Dialister	0.02%	0.00%	0.00%	0.02%	0.00%	0.00%	0.00%	0.00%
Chloronema	0.00%	0.00%	0.04%	0.00%	0.00%	0.00%	0.00%	0.00%
Xylanimicrobium	0.00%	0.00%	0.00%	0.03%	0.00%	0.00%	0.00%	0.00%
Anaerovibrio	0.00%	0.00%	0.00%	0.00%	0.00%	0.00%	0.09%	0.00%
Fusobacterium	0.00%	0.05%	0.00%	0.00%	0.00%	0.00%	0.00%	0.00%
Ignavibacterium	0.00%	0.00%	0.00%	0.02%	0.03%	0.00%	0.00%	0.00%
SMB53	0.02%	0.00%	0.00%	0.00%	0.03%	0.00%	0.00%	0.00%
Guggenheimella	0.00%	0.00%	0.04%	0.00%	0.00%	0.00%	0.00%	0.00%
Mycoplana	0.00%	0.00%	0.04%	0.00%	0.00%	0.00%	0.00%	0.00%
Gemmata	0.00%	0.00%	0.00%	0.02%	0.03%	0.00%	0.00%	0.00%

Anaerobiospirillum	0.00%	0.00%	0.00%	0.00%	0.00%	0.00%	0.09%	0.00%
Cellvibrio	0.00%	0.00%	0.02%	0.00%	0.03%	0.00%	0.00%	0.00%
Paucibacter	0.04%	0.00%	0.00%	0.00%	0.00%	0.00%	0.00%	0.00%
Gemmatimonas	0.00%	0.10%	0.00%	0.00%	0.00%	0.00%	0.00%	0.00%
Acidaminococcus	0.00%	0.00%	0.04%	0.00%	0.00%	0.00%	0.00%	0.00%
rc4-4	0.00%	0.00%	0.04%	0.00%	0.00%	0.00%	0.00%	0.00%
Azohydromonas	0.00%	0.00%	0.02%	0.00%	0.00%	0.00%	0.00%	0.02%
Anaerofilum	0.00%	0.05%	0.00%	0.00%	0.03%	0.00%	0.00%	0.00%
Oscillochloris	0.02%	0.05%	0.00%	0.00%	0.00%	0.00%	0.00%	0.00%
Shinella	0.02%	0.00%	0.00%	0.00%	0.03%	0.00%	0.00%	0.00%
Giesbergeria	0.00%	0.00%	0.02%	0.00%	0.00%	0.00%	0.00%	0.00%
Pseudoramibacter_Eubacterium	0.02%	0.00%	0.02%	0.00%	0.00%	0.00%	0.00%	0.00%
Atopobium	0.00%	0.00%	0.04%	0.00%	0.00%	0.00%	0.00%	0.00%
Lewinella	0.00%	0.00%	0.00%	0.00%	0.05%	0.00%	0.00%	0.00%
Trichococcus	0.00%	0.00%	0.04%	0.00%	0.00%	0.00%	0.00%	0.00%
Geothrix	0.00%	0.00%	0.00%	0.00%	0.00%	0.00%	0.00%	0.02%
Thiomonas	0.02%	0.05%	0.00%	0.00%	0.00%	0.00%	0.00%	0.00%

Supporting material 3

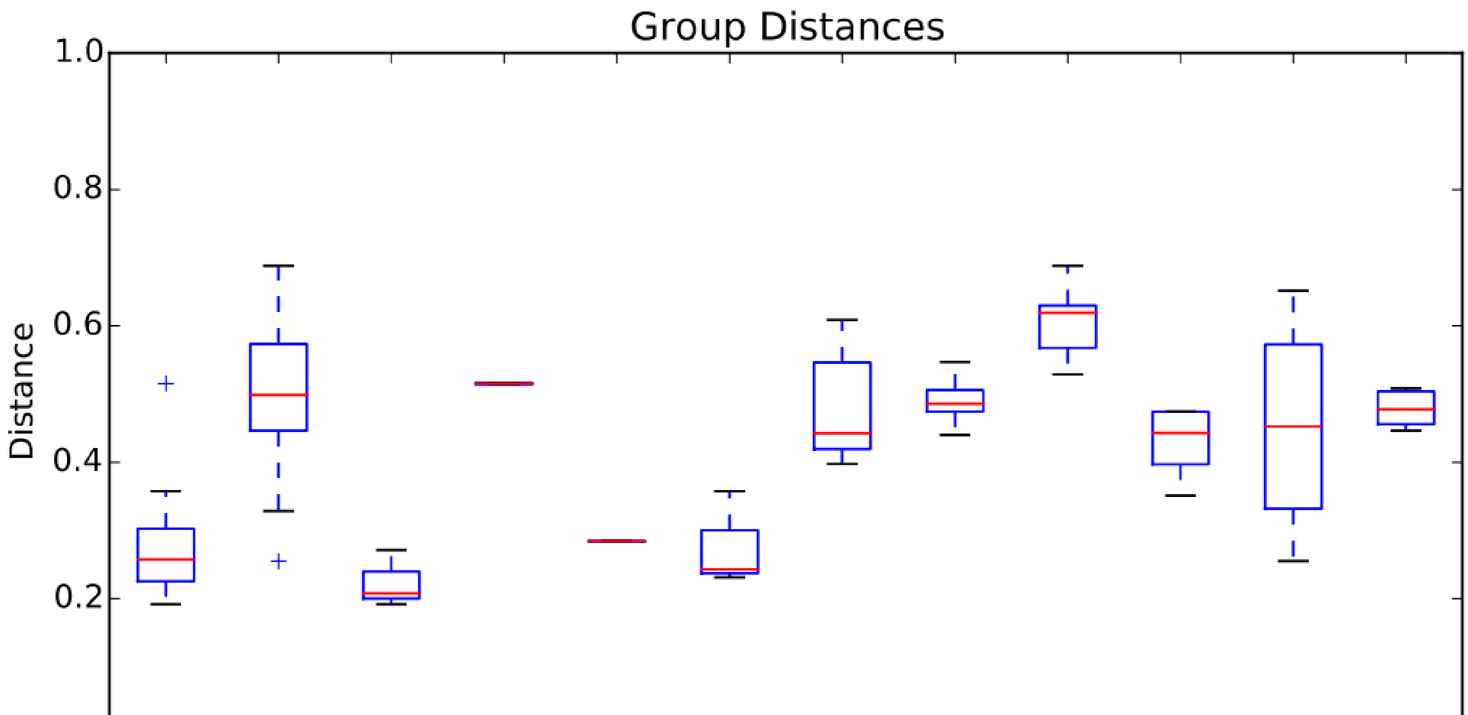

Supplement: Supplementary file 1 — supporting material files [file 41598_2018_22683_MOESM1_ESM.pdf]
